# ReQuant: improved base modification calling by k-mer value imputation

**DOI:** 10.1093/nar/gkaf323

**Published:** 2025-05-10

**Authors:** Roy Straver, Carlo Vermeulen, Joe R Verity-Legg, Marc Pagès-Gallego, Dieter G G Stoker, Alexander van Oudenaarden, Jeroen de Ridder

**Affiliations:** Center for Molecular Medicine, University Medical Center Utrecht, 3584 CX Utrecht, The Netherlands; Oncode Institute, 3521 AL Utrecht, The Netherlands; Center for Molecular Medicine, University Medical Center Utrecht, 3584 CX Utrecht, The Netherlands; Oncode Institute, 3521 AL Utrecht, The Netherlands; Oncode Institute, 3521 AL Utrecht, The Netherlands; Hubrecht Institute-KNAW (Royal Netherlands Academy of Arts and Sciences), 3584 CT Utrecht, The Netherlands; Center for Molecular Medicine, University Medical Center Utrecht, 3584 CX Utrecht, The Netherlands; Oncode Institute, 3521 AL Utrecht, The Netherlands; Center for Molecular Medicine, University Medical Center Utrecht, 3584 CX Utrecht, The Netherlands; Oncode Institute, 3521 AL Utrecht, The Netherlands; Oncode Institute, 3521 AL Utrecht, The Netherlands; Hubrecht Institute-KNAW (Royal Netherlands Academy of Arts and Sciences), 3584 CT Utrecht, The Netherlands; Center for Molecular Medicine, University Medical Center Utrecht, 3584 CX Utrecht, The Netherlands; Oncode Institute, 3521 AL Utrecht, The Netherlands

## Abstract

Nanopore sequencing allows identification of base modifications, such as methylation, directly from raw current data. Prevailing approaches, including deep learning (DL) methods, require training data covering all possible sequence contexts. These data can be prohibitively expensive or impossible to obtain for some modifications. Hence, research into DNA modifications focuses on the most prevalent modification in human DNA: 5mC in a CpG context. Improved generalization is required to reach the technology’s full potential: calling any modification from raw current values. We developed ReQuant, an algorithm to impute full, k-mer based, modification models from limited k-mer context training data. ReQuant is highly accurate for calling modifications (CpG/GpC methylation and CpG glucosylation) in Lambda Phage R9 data when fitting on ≤25% of all possible 6-mers with a modification and extends to human R10 data. The success of our approach shows that DNA modifications have a consistent and therefore predictable effect on Nanopore current levels, suggesting that interpretable rule-based imputation in unseen contexts is possible. Our approach circumvents the need for modification-specific DL tools and enables modification calling when not all sequence contexts can be obtained, opening a vast field of biological base modification research.

## Graphical Abstract

**Graphical Abstract:**
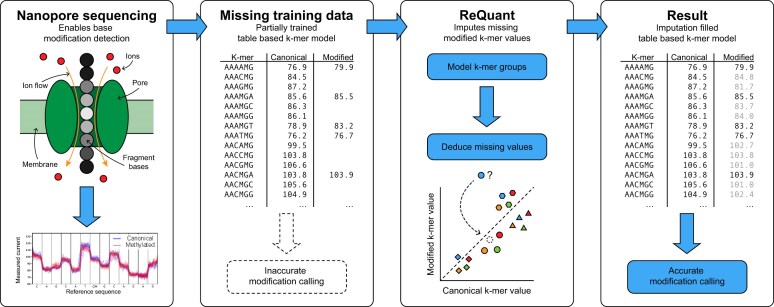

## Introduction

Oxford Nanopore Technologies (ONT) introduced the first commercially available nanopore sequencing device, the MinION, in 2014 [1]. Nanopore sequencing relies on a motor protein ratcheting a DNA (or RNA) molecule through a nanoscale protein pore whilst measuring an ionic current that simultaneously flows through it. While translocating, the DNA strand residing inside the pore disturbs the ionic current in a way particular to its structure. This current value is then used to identify the exact sequence of nucleic acids that flowed through the pore [2]. Due to the length of the sensing domain of the pore, multiple nucleotides at a time influence the measured currents. For ONT’s R9 pore most basecalling tools assume that 5-mers or 6-mers exert a measurable effect on the signal [3–9], whereas for the R10 pore this increases to 9-mers. To convert the measured currents to base sequences, a range of machine learning-based basecallers may be used to determine what sequence of overlapping k-mers is most likely to have caused the measured series of current values [7–11].

DNA modifications greatly expand the genetic code and play a major role in regulation of gene expression and cellular processes. For instance, 5-methylcytosine (5mC) is a modification in which a methyl group is attached to the fifth carbon atom of a cytosine nucleotide. 5mC is most prominently observed in a CpG context (5mCpG) but also occurs in other contexts [12, 13]. While 5mCpG is the most frequent and well-studied DNA modification, it is only one of many known DNA [14–16] and RNA [17] modifications that occur in a range of sequence contexts (e.g. 5hmC, 5fC, 5caC, 6mA, and 4mC). Furthermore, unnatural modifications and bases can be introduced in the laboratory but require models to identify them correctly.

As DNA base modifications alter the current values measured by nanopore sequencing this technology permits detection of DNA (and RNA) modifications directly rather than through chemical or enzymatic methods such as bisulfite conversion [18]. To enable this, various tools have been developed that use the nanopore current values for modification calling [19], most of them limited to specific sequence contexts. The three most commonly used methods for modification calling are (i) basecalling error-based, where the basecalling errors are used to identify noncanonical bases, (ii) table-based, where sequences of raw current values are scored for their similarity to either modified or unmodified expected values, and (iii) Deep Learning (DL)-based, where large collections of training data are used to train sequence-to-sequence models end-to-end [11, 20].

Basecalling error-based modification calling methods do not depend on raw current data directly [6, 21], but use features derived from basecalling errors to predict modifications. This has two downsides: (i) modification detection directly depends on the basecalling algorithm used, and (ii) basecaller improvements that reduce error rates effectively work against these methods. Such methods are hence not future-proof. Other tools such as Tombo also implement a simple statistical approach where significant deviations from the expected canonical current values indicate a modification. While these strategies (partially) circumvent the need for training data, they cannot differentiate between different types of modifications and can be inaccurate [22].

Most of the currently popular modification callers rely on DL to either differentiate between canonical bases and modified bases or directly identify modified bases by expanding the alphabet of possible outputs of the model. DL-based methods are trained on raw canonical and modified current value examples to gain an understanding of their differences. Ideally, DL-based modification callers should generalize to unseen sequence contexts. In practice, however, DL-based models often fail to generalize in unforeseen ways [23].

Further, while DL models are quickly advancing in performance, they are considered black boxes, meaning that their inner workings are not interpretable. The pragmatic approach to their use is to extensively validate models and apply them in practice when performance is satisfactory. When their performance is poor, however, troubleshooting them is challenging and developers are left with adjusting the model architecture, hyperparameters or expanding the training dataset, and hoping that the model will then improve, without learning why the model failed. Additionally, the model’s opacity precludes interpretations which could yield valuable mechanistic insights for further developments and new applications.

Prior to the presently used DL methods, table-based modification calling methods were widely used. Table-based modification calling methods use k-mer tables, in which the expected current value of each possible k-mer is derived from training data in both the canonical and modified state. Populated tables are subsequently used to call modifications by comparing the likelihood that an observed current signal is derived from a sequence with and without a modification. Nanopolish [3], e.g. leverages Hidden Markov modeling for this task. Raw current tables are therefore human-interpretable but may also have missing values for k-mers that were not observed in training, leading to mistakes and uncertain calls [24].

Both table-based and deep-learning methods require training data to develop and validate the modification calling model. At present, such ground truth data should cover all possible sequence contexts in which the modification may occur (and, for some tools, also the canonical alternative). To generate such ground truth data, one option is to enzymatically modify template DNA. This is only possible in contexts for which modifying enzymes exist [22, 25]. Moreover, imperfect enzymatic reaction efficiency can severely affect the resulting ground truth data, and enzyme motif preferences and off-target conversions can introduce sequence biases and noise that confound the resulting model. Alternatively, oligomers can be synthesized with a wide range of known modified bases. Problematically, especially when dealing with the exponential increase in training data needed for longer pore sensing domains (which lead to longer k-mers that influence the signal) and shorter modification motifs (leading to more unique k-mer contexts), generating complete libraries of such oligomers is prohibitively expensive.

Thus, currently, training reliable, table-based or deep-learning models is only feasible for a small subset of the possible DNA modifications where training data covering every sequence context can be produced.

In conclusion, an interpretable, comprehensive approach for all possible modifications is important but lacking.

In this work we present ReQuant, a novel approach to reliably derive k-mer tables for modified bases from limited training data that can be readily employed in table-based modification calling methods. We show that shifts in the current values caused by a DNA modification are shared among similar k-mers, and that it is possible to extrapolate a complete model from a very limited set of k-mer observations. This is in stark contrast to DL-based basecallers, which, as we show for Remora/Megalodon, the current ONT-developed state-of-the-art in DL-based modified base calling, do not generalize well to previously unseen base sequence contexts when trained on a very limited subset of example contexts. We benchmark ReQuant on CpG methylation, a modification for which highly performant models are available, and validation data are easily obtainable, for both R9 and R10 pore versions. We further demonstrate the utility of the ReQuant approach by deriving an accurate R9 pore model for the less well-studied GpC methylation, as well as for glucosylated cytosines. Our approach enables affordable creation of models for modification calling, including rare modifications, where a complete ground truth data set is difficult or too expensive to obtain, and reduces the need for acquiring new training data after kit or software updates.

## Materials and methods

### Data preparation

Nonmethylated Lambda Phage DNA (Sigma–Aldrich, D3654) was used.

#### Generation of ^m^CpG Lambda Phage genome

Individual 20 μl of methylation reactions were set up as follows: 1 μg of nonmethylated Lambda Phage DNA (Sigma–Aldrich, D3654), 20U CpG Methyltransferase M.SssI (NEB, M0226L), 1× NEBuffer 2 (NEB, B7002S), 0.8 mM S-adenosylmethionine (SAM) (NEB, B9003S). Samples were incubated for 24 h at 37°C with an additional 0.8 mM SAM spiked in at 4 and 8 h. Samples were cleaned up as follows: 0.12 U Thermolabile Proteinase K (NEB, P8111S) was added to each reaction and incubated at 37°C for 2 h then 55°C for 10 min. 22L (1× volume) H_2_O was added followed by 44L (1× volume) AMPure XP beads (Beckman Coulter, A63882). Elution was performed at 50°C for 10 min.

#### Generation of Gp^m^C Lambda Phage genome

Individual 50 μl of methylation reactions were set up as follows: 1 μg of nonmethylated Lambda Phage DNA (Sigma–Aldrich, D3654), 20U GpC Methyltransferase M.CviPI (NEB, M0227), 1× GC Reaction Buffer (NEB, B0227S), and 0.8 mM SAM (NEB, B9003S). Samples were incubated for 2 or 4 h at 37°C and cleaned up as for CpG genome.

#### Generation of gluC Lambda Phage genome

Methylated genome was glucosylated using NEBNext Enzymatic Methyl-seq Conversion Module (NEB, E7125). 500 ng of genomic DNA (gDNA) in 15 μl was mixed with: 10 μl of reconstituted TET2 reaction buffer (NEB, E7126AAVIAL, and E7126AAVIAL), 1 μl of Oxidation Supplement (NEB, E7128AAVIAL), 1 μl of 100 mM DTT (NEB, E7139AAVIAL), 3 μl of Oxidation Enhancer (NEB, E7129AAVIAL), 12 μl of TET2 (NEB, E7130AAVIAL), 20U T4 Phage-glucosyltransferase (T4-BGT) (NEB, M0357), and 40 μM uridine disphosphate glucose (UDP glucose) (NEB, B7004). Next, 5 μl of diluted Fe (II) Solution (0.4 mM) (NEB, E7131AAVIAL) was added. Samples were incubated at 37°C for 3 h before addition of 1 μl of Stop Reagent (NEB, E7132AAVIAL) and further incubation at 37°C for 30 min. Samples were cleaned as above.

#### Library prep

Library preparation was performed using SQK-LSK109 (Oxford Nanopore) and samples were sequenced on an Oxford Nanopore GridION X5 Mk1 with Flongle R9.4.1 flow-cells (FLO-FLG001) and MinKNOW (v21.05.25, v21.2.5, v22.3.4).

#### CliveOME

We downloaded ONLA29132 (fast5 files) and the matching PAM63167 (bam file with Bonito’s modcalling results) from the CliveOME dataset (https://labs.epi2me.io/cliveome_5mc_cfdna_celldna/). This dataset contains human cell-free DNA reads produced by ONT and includes Bonito methylation calls. We extracted fastq files from the bam file using Samtools. Using modkit 0.2.3 we created a bedmethyl file to use as ground truth for training Nanopolish, and a modcall file as ground truth for testing the calls per read.

### Remora/Megalodon

DL models were trained using a combination of Megalodon, Taiyaki, and Remora. A separate reference fasta file per was created and used to map and train signals. Steps taken to process data are: *megalodon signal_mapping*, *taiyaki merge*, *remora prep*, *remora train*, and *megalodon call* (see Supplementary data: Remora/Megalodon).

### R9: Nanopolish

At the time of writing ReQuant’s algorithm, the R10 branch of Nanopolish was suggested for training new models. We based our work here on commit “9681478e87c2f8e54e9aab994c73572a20f3ae98.”

We prepared an expanded model using “expand_model_alphabet.py” found here: https://github.com/jts/methylation-analysis. This script takes the canonical k-mer model and generates all possible k-mers with ATCGM, where the M marks the modified base. Values for each newly generated k-mer are copied from the canonical equivalent k-mer (replacing any M with a C) while standard deviations are copied and increased by 1. To make GpC k-mer tables we made minor alterations to Nanopolish (see Supplementary data: Nanopolish edits).

### R10: f5c

The R10 pore version supported by Nanopolish appeared to have been an earlier version and processing data requires several edits. Instead, we roughly followed the guide from f5c: https://hasindu2008.github.io/f5c/docs/r10train#nucleotide-model.

We used their R10 template model (r10.4.1_400bps.cpg.9mer.model) for training and their precompiled fork of Nanopolish to train a model. The f5c tool was used to call methylation for R10 data.

**Figure 1.**
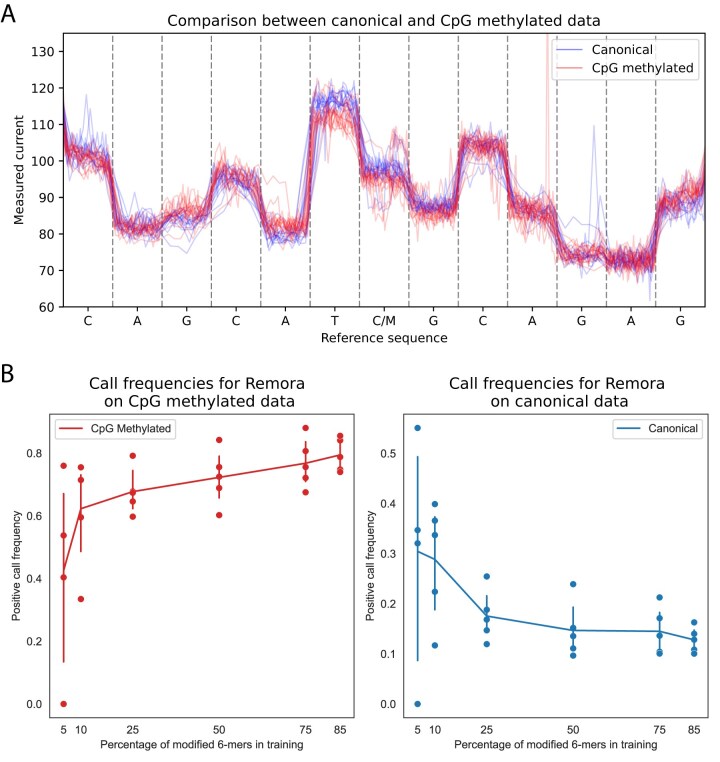
(**A**) Raw currents for Lambda Phage genome data aligned to the matching reference sequence (20614–20626), showing individual reads with canonical bases in blue (fully unmethylated; see Materials and methods) and reads with modified bases in red (fully methylated; see Materials and methods). The difference in observed current values is not limited to the modified base (marked as C/M in the center), surrounding bases are affected as well. (**B**) True positive call frequencies on CpG-methylated data (left) and false positive call frequencies on canonical data (right) when training Remora on various sections of the Lambda genome, containing the percentages of modified 6-mers shown on the *x*-axis, and calling on other sections using Megalodon. Error bars represent bootstrapped 95% confidence intervals of the mean.

**Figure 2.**
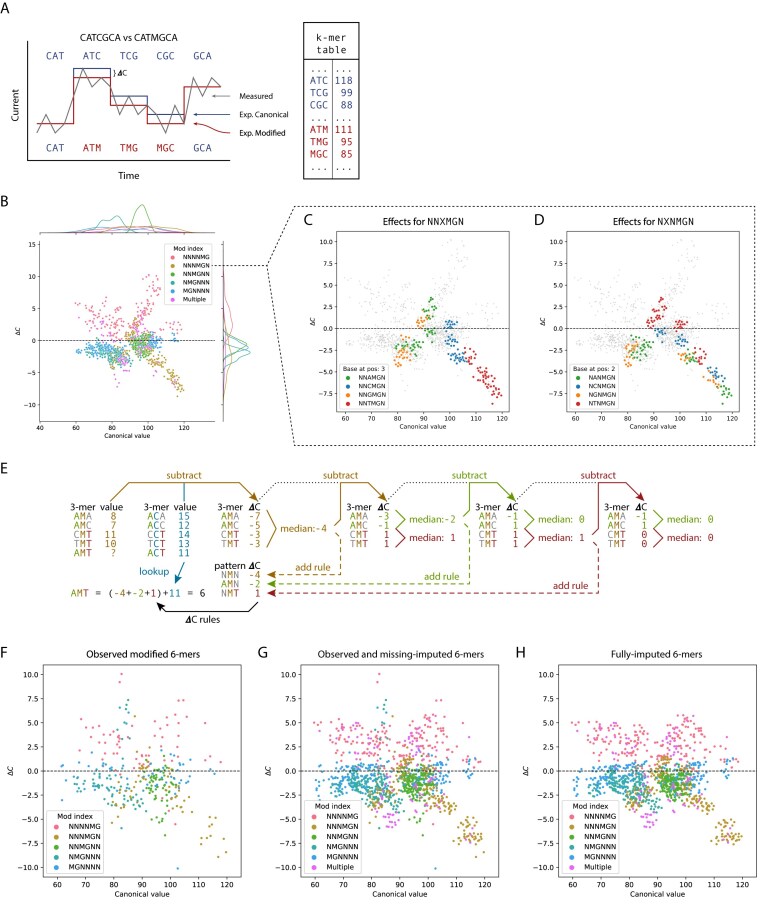
(**A**) Simplified cartoon of Nanopolish and Tombo table-based modification calling: an observed raw current value is compared to expected current values for both canonical and modified sequences. The likelihood of the sequence of current values being emitted by either is calculated and the most likely sequence to have caused the observed values is called. (**B**) Scatter plot showing the expected current of CpG containing 6-mers (*x*-axis), and the expected difference in current for CpG-methylated 6-mers (ΔC, *y*-axis), according to the CpG 6-mer table included with Nanopolish. Colors indicate the position of the MG motif in the modified 6-mers. (**C** and **D**) Scatter plots as in panel (B), but for 6-mers with the modification motif at the fourth position (NNNMGN), colored by the base at the third position (NNXMGN, C), and for the base in the second position (NXNMGN, D). (**E**) Simplified example of determining ΔC rules and applying them to obtain the unknown, expected modified current value for the AMT 3-mer. The first ΔC column shows the difference between a modified 3-mer and its canonical counterpart (e.g. AMA-ACA) for the training 3-mers. The ΔC rules are determined from these differences by taking the median of all matching patterns, after subtracting the values of applicable, previously determined rules, if any [e.g. AMN is the median of AMA and AMC after subtracting NMN for both (i.e. AMA: −7 − (−4) = −3; AMC: −5 − (−4) = −1; median of AMN: (−1 + (−3))/2 = −2, which becomes the ΔC rule for AMN)]. Finally, the relevant rules are summed and added to the canonical equivalent of the 3-mer to be imputed (here, assume AMT has not been seen in the training data). (**F**) Expected current value differences measured for methylated 6-mers in a Nanopolish CpG model after training in a *T*= 25% run. That is, 25% of unique modified 6-mers were minimally sampled from a fully methylated Lambda Phage genome. Only 6-mers that were trained on are shown. Canonical values are shown on the *x*-axis, while current differences caused by their modification are shown on the *y*-axis. Colors indicate the location of the CG motif in the 6-mers. (**G**) The same plot as panel (F), but the missing 6-mer values not included in training are now imputed through ReQuant and plotted as well. Note how the spread of 6-mer values resembles the distribution of values in panel (B). (**H**) Same plot as panel (G), but the 6-mers that were originally trained have been imputed by ReQuant (essentially refining the Nanopolish 6-mer table values using information from similar 6-mers).

### ReQuant algorithm

ReQuant’s implementation is shown schematically in Supplementary Fig. S1. While this implementation is different as it is optimized for speed, it should provide the same output as the algorithm outlined here. For this algorithm, we assume a fully trained canonical k-mer table is available, and a set of k-mers of the same length with a modified base has already been trained (i.e. the expected current value per k-mer is known).

Let ${{\bf x}}$ be a k-mer of length $k$, e.g. for R9 models it is commonly assumed 6 bases influence the signal, i.e. $k$=6. Let ${\boldsymbol{X}} = \{ {{{{\bf x}}}_1,{{{\bf x}}}_2,\ldots} \}$ be the set of all k-mers with a potential modification, e.g. ${\boldsymbol{X}}$ contains only k-mers with a CG motif for CpG methylation. Let ${{\bf v}} = \{ {{v}_1,{v}_2,\ldots} \}$ be the associated canonical signal levels, let ${{\bf \hat{v}}} = \{ {{{\hat{v}}}_1,{{\hat{v}}}_2,\ldots} \}$ be the associated signal levels of the modified counterparts and let ${\boldsymbol{\Delta }}{{\bf v}} = {{\bf v}} - {{\bf \hat{v}}}$ be the observed signal differences. Let ${\boldsymbol{T\ }}$be the set of ‘training’ k-mers, i.e. all $i$ for which both ${v}_i$ and ${\hat{v}}_i$ are known.

Let ${{\boldsymbol{I}}}_m{\boldsymbol{\ }}$be the set of k-mers where position $m$ can become modified, e.g. ${\boldsymbol{X}}( {{{\boldsymbol{I}}}_4} ) = \{ {\text{``}{\mathrm{NNNCGN}}\text{''}} \}$ where N is any of the four canonical bases. Let ${{\boldsymbol{I}}}_{f,b}$ be the set of all k-mers where the base at position $f \in [ {1,k} ]$ is $b \in \{ {^{\prime}{\mathrm{A}}^{\prime},^{\prime}{\mathrm{C}}^{\prime},^{\prime}{\mathrm{T}}^{\prime},^{\prime}{\mathrm{G}}^{\prime}} \}$, e.g. $\text{``}{\mathrm{NTNNNN}}\text{''}$ for $f = 2$, $b = {\mathrm{^{\prime}T^{\prime}}}$. Let $r > 0$ be a predefined stopping threshold, e.g. default $r = 0.01$, but this value depends on scaling of the input table.

The estimated signal value ${\hat{v}}_i$ for a modified k-mer ${{{\bf x}}}_i$ can now be calculated as follows:

- Define ${\boldsymbol{I}} = {\boldsymbol{T}} \cap {{\boldsymbol{I}}}_m$ for $m$ equal to the position of potential modification in ${{{\bf x}}}_i$ and initialize:
  \begin{eqnarray*}
{\hat{v}}_i\ = {v}_i + {\mathrm{median}}( {{{\bf \Delta v}}( {\boldsymbol{I}} )} )
\end{eqnarray*}
- Correct the difference vector:
  \begin{eqnarray*}
{{\bf \Delta v}}( {\boldsymbol{I}} ) = {{\bf \Delta v}}( {\boldsymbol{I}} ) - {\mathrm{median}}( {{{\bf \Delta v}}( {\boldsymbol{I}} )} )
\end{eqnarray*}
- With $m$ equal to the position of a potential modification in ${{{\bf x}}}_i$, determine ${f}^*$ and ${b}^*$ for which:
  \begin{eqnarray*}
&{f}^*,{\mathrm{\ }}{b}^* = {\mathrm{arg}}\mathop {\max }\limits_{f,b} | {{\mathrm{median}}( {{\mathrm{\Delta }}{{\bf v}}( {{\boldsymbol{T}} \cap {{\boldsymbol{I}}}_m \cap {{\boldsymbol{I}}}_{f,b}} )} )} |\\ &{h}^* = {\boldsymbol{\ }}{\mathrm{median}}( {{{\bf \Delta v}}( {{\boldsymbol{T}} \cap {{\boldsymbol{I}}}_m \cap {{\boldsymbol{I}}}_{{f}^*,{b}^*}} )} )
\end{eqnarray*}
- If $| {{h}^*} | > r$, adjust as:
  \begin{eqnarray*}
&{{\boldsymbol{\hat{v}}}}_i\ = {{\boldsymbol{\hat{v}}}}_i + {h}^*\\ &{{\bf \Delta v}}( {\boldsymbol{I}} ) = {{\bf \Delta v}}( {\boldsymbol{I}} ) - {h}^*
\end{eqnarray*}
- Repeat steps 3–4 until $| {{h}^*} | \le r$

For a k-mer with multiple modified bases (e.g. *X*=“MGMGMG”) the algorithm is run for all positions with a modified base. For the subsequent modified bases the value of ${{v}_i}$ in step 1 is replaced by the final value of ${{\hat{v}}_i}$ in the previous run.

In ReQuant’s implementation, k-mers with the modification in an incomplete motif (e.g. at the end and missing the G of the MG motif in “NNNNNM”) are left out. While ReQuant does work with these included, we observed no significant shifts applied to these groups. Including them would significantly increase the total number of possible modified k-mers (from 1280 to 2304) without adding any actual information for calling modifications. This would decrease the percentages of k-mers necessary for training simply by padding the number of possibilities with uninformative data, providing potentially misleading results. Hence, we left them out.

## Results

### Existing modification calling strategies do not accurately identify DNA modifications in sequence contexts not observed in training

The raw signal produced in Nanopore sequencing is a sequence of noisy current measurements. The combination of multiple bases (k-mer) in a pore at any time influences the current values. As a nucleic acid fragment travels through the pore at variable speeds, the number of measurements per k-mer varies, forming measurement segments of variable length, with similar current values. Measured current values in a segment are different for k-mers containing one or more modified bases (Fig. 1A). Modification-calling using raw Nanopore currents attempts to determine whether a series of current values is more similar to the expected segment values for a canonical sequence, or segment values for a modified sequence.

We trained Remora/Megalodon (R/M), a leading DL method, on subsets of 6-mers, simulating situations wherein the model has access to only a limited set of 6-mer sequence contexts during training. We applied these R/M models to a dataset containing (i) only methylated CpG sites (Fig. 1B, left panel) or ii) only unmethylated CpG sites (Fig. 1B, right panel). As the percentage of unique 6-mers provided in training decreases, R/M calls fewer methylated 6-mers as methylated (fewer true positives) and more unmethylated 6-mers as methylated (more false positives). Furthermore, the spread in true- and false-positive call frequencies indicate a lack of robustness (Fig. 1B, Materials and methods: Remora/Megalodon). Consequently, we find that these deep-learning models do not generalize well when applied to 6-mer contexts not provided in the training data.

Table-based methods, such as Nanopolish [3], f5c [26], and Tombo [22], work with a 1-to-1 mapping between a k-mer and an expected current value. If the expected current value of a modified sequence is not present in the table it cannot be called. Table-based methods are therefore also unable to extrapolate to k-mers outside of the training data (Fig. 2A, Materials and methods: Table-based methods).

As a result, we find that presently available modification calling tools cannot accurately determine the modification status of DNA contexts not present in the training data, thus requiring complete training data sets. As this is often prohibitively difficult to obtain this limits future research studying the vast array of endogenous, or using artificially introduced, nucleic acid modifications.

### Tables of expected current for (un)modified k-mers can be summarized as ΔC rules

We propose ReQuant, a method to predict expected Nanopore current values of modified k-mers never-observed in training, using information shared from similar k-mers. We achieve this by focusing on the current value difference between a modified k-mer and its canonical equivalent observed in training and projecting this difference to similar k-mers not observed in training. These current value differences are easily accessible in the k-mer table of a typical table-based method, such as the 6-mer CpG methylation model of Nanopolish (Fig. 2A).

To understand which k-mers show similar expected current differences upon modification we used the expected current table provided with Nanopolish to examine the difference between expected current values of canonical and modified 6-mers (which we henceforth refer to as ΔC).

First, we found that k-mers with a modification motif in the same location within a k-mer show similar current shifts on modification, indicating some structure dictated by the position of the modification in the 6-mer (Fig. 2B, points of the same color cluster together).

Second, we found that, for k-mers with a modification motif in the same location, k-mers with the same base immediately adjacent to the modification motif show similar current shifts on modification (Fig. 2C, points of the same colour cluster together). For instance, 6-mers with the sequence “NNTMGN” (N meaning any base, M denoting the modified base) form a pronounced cluster in the plot (Fig. 2C, red dots). Third, we further subdivided NNTMGN k-mers into 6-mers with the same base two nucleotides away from the modification motif (NXTMGN). We found, again, that k-mers with the same base two nucleotides from the motif show similar current shifts on modification, but that this similarity is less pronounced than for k-mers with the same base 1 nucleotide away from the motif (Fig. 2D). Thus, we observe that k-mers with similar structures show similar current shifts on modification and begin to elucidate logical rules governing these current shifts.

Given these observations, we hypothesized that the entire range of expected modified k-mer current values could be be predicted from limited training data using a limited set of context-dependent current value changes (which we call ΔC rules) that convert canonical current values to modified current values. ReQuant deduces these ΔC rules from observed training data, on a limited subset of k-mers, using a stepwise process (see Materials and methods for details). Subsequently, the ΔC rules are applied to the canonical k-mer table to yield a complete modified k-mer table including k-mers that were unobserved in training and were, therefore, not used to determine the ΔC rules. In effect, we can impute current values for modified k-mers not observed in training using information from similar modified k-mers that are observed.

This principle is shown in Fig. 2E for a simplified toy example, where the aim is to estimate the current value for the “AMT” 3-mer from its unmodified counterpart (“ACT”) and known, partially overlapping 3-mers. First, ΔC values are computed for the 3-mers for which both canonical and modified current values are known (observed in training) by taking the difference between a modified 3-mer and its canonical counterpart (e.g. AMA-ACA). Based on the ΔC values, ΔC rules can be determined that describe the change in current caused by different patterns in the 3-mer. This is achieved by taking the median of all ΔC values that match a certain 3-mer pattern. The first rule estimates the effect of the position of the modification itself, which for the modification in the center position (“NMN”) is −4. Next, the effect of other patterns is evaluated. When k-mer patterns are nested, the pattern with the largest effect is applied first (see Materials and methods). After correcting for “NMN” by subtracting −4, the median for “AMN” is −2 and “NMT” is 1, respectively. Since the median effect for “AMN” is larger, the rule for “AMN” is determined and applied second, after the first rule describing the effect of the position of the modification itself. This process is applied iteratively, until all pattern medians fall below a preset threshold (0.01). In the toy example, the final rule is + 1 for “NMT.” As no 3-mer pattern with a ΔC value median > 0.01 can now be found, the algorithm stops. To determine the expected modified k-mer current value from the canonical value afterward, the relevant rules are summed and added to the canonical equivalent. In the example, the estimated modified current value for the 3-mer “AMT” is the expected unmodified current value (“ACT”) plus the relevant ΔC rules (all of them in this case), i.e. 11 + (−4 − 2 + 1) = 6. Unlike in this toy example, in larger k-mer tables not every rule applies to every k-mer.

### k-mer tables can be summarized as ΔC rules

As a proof of concept, we ran ReQuant on the complete CpG methylation model table Nanopolish uses by default. Here, all values in the table are known and can thus be used to establish the accuracy of ReQuant’s predictions.

In the first iteration of ReQuant, we find that modification of the CG motif in the first position (MGNNNN), second position (NMGNNN), to the fifth position (NNNNMG), caused a median expected current value difference of −0.85, −1.99, −0.66, −2.33, and 2.97, respectively (Supplementary Fig. S2). After applying these shifts to the values in the table, ReQuant then tries to iteratively explain the residual current deviations using other patterns in the 6-mers. The largest ΔC rule found had mC in the 4th position and a T on the third position, creating the ΔC rule (NNTMGN: −4.10). This rule was then applied to the values in the table, and the next largest ΔC rule was determined (i.e. NTNMGN: 3.12). After 69 such ΔC rules were established, no further 6-mer pattern showed a deviation >0.01 and the set of rules was completed. Using this strategy, the expected values for all 1280 6-mers with a single methylated CpG motif were summarized in just 69 ΔC rules. We then recreated all modified 6-mer values by applying the relevant ΔC rules to the canonical current values (Supplementary Fig. S3). Creating the modified k-mer expected current table entries exclusively by applying the ΔC rule set to the unmodified table entries produced a highly similar model to the original Nanopolish model (Supplementary Fig. S3C). We conclude that there are indeed consistent patterns in the expected ΔC values, caused by methyl-cytosine, within similar k-mers and that ReQuant can accurately summarize these as a small collection of ΔC rules.

### ΔC rules enable ReQuant to impute expected current values for k-mers missing from training data

We hypothesized that it is only required to know a fraction of the canonical-modified k-mer current differences from training to establish a functional set of ΔC rules. Thus, we sought to apply ReQuant on incomplete training data covering only a limited number of sequence contexts. To achieve this, we sequenced Lambda Phage genome samples enzymatically modified from 100% negative to near 100% positive cases for the following modifications: CpG Methylation (5mCpG), GpC methylation (5mGpC), and CpG Glucosylation (5GlucCpG). The 50 kilobase Lambda Phage genome contains 100% of all possible 6-mers with a CG motif and 99.8% of all possible 6-mers with a GC motif, making it suitable for testing ReQuant’s imputation performance.

First, we randomly selected CpG sites in the Lambda genome and added the five 6-mers that fully overlap the CpG site (e.g. sequence context TTAAMGAATT yields TTAAMG…MGAATT) until we covered approximately the intended target percentage (*T*) of all possible modified 6-mers (excluding NNNNNM, as we cannot ensure the next nucleotide is a G). We excluded any sites that would not add any new 6-mers to the training set, thus resulting in a near-minimal set of occurrences for the 6-mers included for training (see Materials and methods). We then used Nanopolish to train a new model on a fully methylated (5mCpG) Lamda Phage gDNA sample using only the included sites, creating a 6-mer table where only observed modified 6-mers are adjusted (Supplementary Fig. S4). We repeated this process five times for each setting of *T ∈ {5, 10, 25, 50, 75, 85}* to investigate at what percentage of observed unique modified 6-mers in the training data ReQuant could accurately perform imputation. Note, we did not (need to) train on a negative dataset: the canonical 6-mer table provided by Nanopolish served as a basis and we only trained for the effects of modifications compared to this model. We found that the learned rules stabilize from *T*= 25% onward, as indicated by the reduced spread in the boxplots of ReQuant-determined ΔC rules (Supplementary Fig. S5).

We compared three variations of dealing with 6-mers missing in the training set: not-imputed (Fig. 2F), where unobserved modified 6-mers use the expected current values of their canonical counterparts, missing-imputed (Fig. 2G), where ReQuant imputed the expected current values of modified 6-mers that were absent in the training data using the detected ΔC rules, and fully-imputed (Fig. 2H), where the ΔC rules are not only applied to impute the expected current values of missing 6-mers, but also to overwrite those derived directly from training.

All fully-imputed models produced strongly correlated imputed values for *T*≥ 25% (Supplementary Figs S6 and S7), indicating that at *T*= 25% sufficient training data is obtained to construct reliable ΔC rules. In contrast, models based on fewer training 6-mers (*T*< 25%) had fewer ΔC rules, probably due to a lack of examples (Supplementary Fig. S8A).

Consequently, we concluded that ReQuant was able to reproducibly produce a set of ΔC rules, from training data containing as little as 25% of all possible 6-mers, and accurately impute expected current values for modified 6-mers for all possible 6-mers, from this limited training data.

### Imputed expected current values from ReQuant enable sensitive and accurate CpGme detection with limited training data

**Figure 3.**
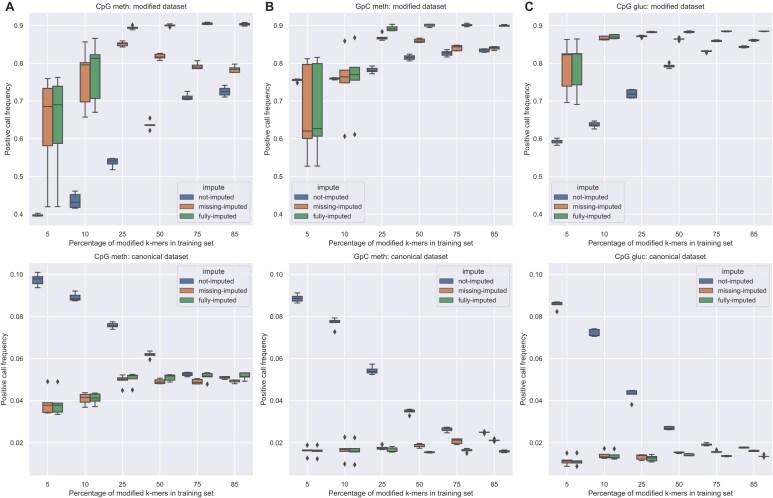
Boxplots showing positive call rates (*y*-axis) on a positive (top; true positive rate) and negative (bottom; true negative rate) dataset, for five randomized repeats of each imputation approach (not-, missing-, and fully-imputed) and per percentage of 6-mers included (*x*-axis) for training on (**A**) CpG methylation data, (**B**) GpC methylation data, and (**C**) CpG glucosyl data.

In order to evidence the efficacy of the imputed expected current value table produced by ReQuant for the desired end use of modification calling we tested the three model imputation variations (not-imputed, missing-imputed, and fully-imputed) using Nanopolish “call-methylation” on two test sets. The first was an independent, CpG-methylated Lambda genome and the second an unmethylated Lambda genome. For each model we ignored calls made at CpG sites that were included for training. However, we did not filter out calls made at other sites that (partially) overlapped the trained 6-mers. This filtering approach best matches a situation where a subset of all possible contexts can be obtained for training. We plotted the positive call rates on the modified set (top row) and the canonical set (bottom row) separately to make trade-offs between false positives and false negatives explicitly visible (Fig. 3A).

We first note that the not-imputed method (i.e. Nanopolish with expected current table values for 6-mers covered in training only) showed lower CpGme calling on fully methylated DNA (more false negatives) and higher CpGme calling on unmethylated DNA (higher false positives). Thus, the not-imputed method, representing standard performance in the absence of complete training data, performed markedly worse than either of the ReQuant-imputed methods. This holds even when most of the 6-mers are included in the training set (*T*= 85%; all possible unique modified 6-mers occuring in the Lambda Phage genome).

The fully-imputed approach performed best. It reaches 90% positive calls on the CpG-methylated genome (true positives) and 95% negative calls on the demethylated genome (true negatives). Further, in agreement with our above findings, ReQuant-based imputation is already effective for training datasets covering as little as *T*= 25% of the modified 6-mers, with little performance improvements observed on addition of more complete training data. Below *T*= 25%, imputation is unreliable: true positive call frequencies vary greatly between different 6-mer samplings (Fig. 3A, top row; large boxplot spread). Combined with the number of false negatives (Fig. 3A, bottom) and the wide boxplot spread at these *T*-values, it appears the imputed models are unreliable at these training percentages. Additionally, we find that if we choose an optimized 6-mer training set ReQuant performs well at only *T*= 16% of 6-mers covered (Supplementary data: Bare minimum training set, Supplementary Fig. S9).

In Nanopolish, a LLR threshold of 2 for calling a base modified or unmodified is used. To ensure that ReQuants seemingly improved accuracy was not a result of a general decrease in LLR’s, resulting in only calling the most easily called loci, we examined the total number of discarded (uncertain) base calls for each imputation model. We show that this is not the case with the fraction of discarded calls largely unchanged or actually decreasing for the fully-imputed model as less training data are used (Supplementary Fig. S10A).

The missing-imputed method performed increasingly worse at *T >*25% in the CpG-methylated genome, suggesting that only imputing 6-mers missing from the training set is insufficient and that overfitting on the 6-mers that do appear in the training set occurs. Because ReQuant integrates the effect of a base modification across similar 6-mers, it is less vulnerable to this overfitting. The observed improvement remains when considering call rates based on sites used in training only (Supplementary Fig. S11A), indicating that the ReQuant-based imputation truly exerts a denoising effect that further improves 6-mer estimates beyond the information in the data belonging to individual 6-mers. In the opposite situation, where only calls without any trained 6-mer overlap are considered, the not-imputed method cannot apply any useful information from the trained 6-mers to improve the call rates (Supplementary Fig. S12A). This observation is limited to models where *T*≤ 50%, as the number of sites in the Lambda genome without any overlapping 6-mers quickly diminishes when the training set size increases (Supplementary Fig. S13A).

In sum, ReQuant clearly improved the expected modified 6-mer current values by combining information from other 6-mers, for both 6-mers observed and unobserved in the training set, in a CpG motif context.

### ReQuant may be extended for efficacious detection of methylation in non-CpG motifs, with limited training data

Given ReQuant’s performance on CpG methylation, we turned to GpC methylation data to show our approach is not motif dependent. Analogous to our CpG imputation we split (enzymatically methylated) GpC sites on a Lambda genome into a training and test set and calculated the set of ΔC rules specific for GpC methylation. Most of the rules determined for the CpG models are not transferable (Supplementary Figs S5 and S14), since the base preceding the modified Cytosine is now consistently a Guanosine, and the base following the methylated Cytosine is now variable. We find that both ReQuant’s fully as well as the missing-imputed models outperformed the not-imputed model for GpC methylation calling (Fig. 3B). Despite a smaller performance difference than in the CpG context, the not-imputed model only reaches its performance by discarding many of its calls for *T*≤ 25% (Supplementary Fig. S10B). Otherwise, we observe the same trends as for the CpG results: the missing-imputed approach decreases at *T >*25% as it cannot compensate for noise in 6-mers used for training, and the fully-imputed approach levels off at *T ≥*25% indicating that there is little left to learn from additional data. However, the negative call frequencies show a lower false positive rate for ReQuant imputation approaches across a broad range of values for *T*. For the missing-imputed approach the false positive rate increases, as it is unable to effectively compensate for noise in the training 6-mers. Similar to the CpG setting, we find that the number of discarded calls for imputed models remains low for *T >*25%, and similar to or lower than not-imputed models at *T*= 85% (Supplementary Fig. S10B). For completeness, call frequency results for exclusively training sites (Supplementary Fig. S11B), sites with no 6-mer overlap with the training data (Supplementary Fig. S12B) and the number of calls included in each plot (Supplementary Fig. S13B) are provided.

### ReQuant performs well when adapted to nonmethylation modifications

Next, we sought to demonstrate that our approach generalizes to other modifications. To this end, we created samples with glucosylated cytosines in CpG sites using enzymes Tet methylcytosine dioxygenase 2 (TET2) and T4 Phage β-glucosyltransferase (T4-BGT). Considering the size of this modification, we expected an extreme ΔC between canonical and modified k-mers. We ran the same analysis as for CpG methylation above, replacing the training and test samples with glucosylated samples. Compared to methylation calling, the ΔC rule values are much larger (Supplementary Fig. S15), indicating that as expected the glucosyl group has a larger effect on measured current values. The not-imputed approach reaches higher positive call frequencies than 5mCpG calling and less false positives when nearing complete 6-mer training sets (Fig. 3C). Remarkably, ReQuant already performs near-optimally at *T*= 10% 6-mer coverage. As before, the results are not due to conservative filtering of the calls (Supplementary Fig. S10C), as ReQuant-discarded call frequencies at *T*≥ 10% are similar to or below not-imputed discarded call frequencies at *T*= 85%. Trained sites show little improvement for *T*> 5% (Supplementary Fig. S11C), while the fully imputed sites reach similar call rates at *T*= 10% (Supplementary Fig. S12C). The sites included for these statistics are the same as for CpG methylation (Supplementary Fig. S13C).

Taken together we find that ReQuant can be extended to non-CpG motifs as well as to modifications beyond just methylation, and, works better if the modification’s impact on the signal is larger.

### ReQuant remains valid for data from the most recent R10 pore model

While current values obtained from R9 pores are well-modeled with 6-mers, R10 pores come with an increased barrel length and a “double reader-head,” meaning that the measured current is affected by >6 bases at a time. This leads to models with longer k-mers, and thus an exponential increase in possible k-mers in the modification table (4^9^ 9-mers for canonical bases alone in R10). As a result, a similarly exponential increase in the required training data for modification calling methods is expected.

We tested ReQuant on R10 data using data obtained from a subset of the human CliveOME 5mC dataset (see Materials and methods for details) combined with the model and training approach from f5c (an alternative for Nanopolish for raw current value alignment and modification calling, see Materials and methods: R10: f5c). We extracted the included modification calls (provided by Bonito, a DL-based modification caller) from the CliveOME dataset to use as ground truth. As the CliveOME 5mC R10 dataset was fully produced by ONT, the maker of the nanopore sequencers, this ensures the data was prepared and processed optimally for the platform and should be among the most reliable methylation call data available, providing a purely technological reference for our methods. We trained a not-imputed model on Chromosome 22 using a modified version of Nanopolish. This resulted in training ∼36% of all possible modified 9-mers.

Using ReQuant on this not-imputed model we created both a missing-imputed and a fully fully-imputed, using 207 generated ΔC rules (Supplementary Fig. S16). We observe that the strongest current value difference is in 9-mers with the modification at position 6 or 7. This corresponds to when the modified base just enters the pore, and indicates that the (modified) bases in the first bottleneck have by far the most important effect on current flow, and that the second bottleneck in R10 pores does not add much information here. ReQuant's full imputation drastically reduces the large spread in k-mer ΔC values seen in the not-imputed situation (Supplementary Fig. S17). All models were tested on Chromosome 21 using f5c (see Supplementary Fig. S18 and Materials and methods for details).

**Figure 4.**
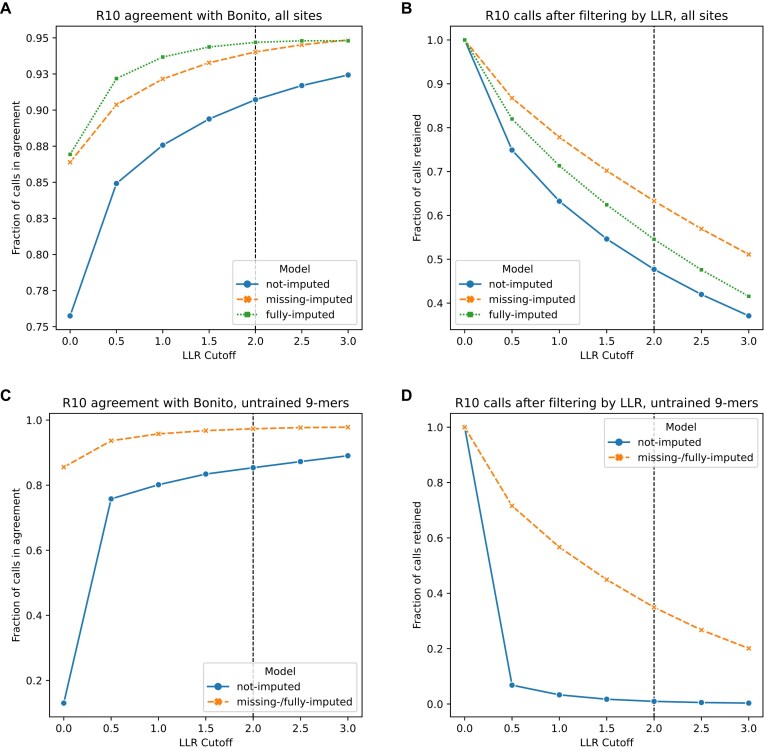
Plots showing the agreement between the Bonito methylation calls included in the CliveOME dataset and the Nanopolish/f5c model that was trained on chromosome 22 and tested on chromosome 21 from the same CliveOME dataset. The vertical line at *x* = 2 indicates the usual cutoff for Nanopolish results. (**A**) Agreement for all sites on chromosome 22. (**B**) The total number of calls retained after filtering at various LLR cutoff values. At *x* = 0, no filtering happens, and the upper limit is shown. This number indicates how many sites were called in both Bonito and Nanopolish/f5c. (**C**) Similar to panel (A), but only including calls made on sites without overlap with any trained 9-mer, thus focusing on the imputed data only. (**D**) Similar to panel (B), but as with panel (C), only including sites without overlap with any trained 9-mers. In both panels (C) and (D), the missing- and fully-imputed models are shown as one line, since filtering out all sites that overlap trained 9-mers left both models with the same set of imputed 9-mers, and hence, the same calls.

Compared to the not-imputed model, the R10 models produced by ReQuant show improvement in both precision (agreement between ReQuant/f5c and Bonito calls) and recall (the number of calls kept after filtering at various cutoff values of the LLR) (Fig. 4A and B). The improvement is relatively small, which may be due to similar, nonuniform, 9-mer occurrence rates between chromosome 21 and chromosome 22 (Supplementary Fig. S19). Such similarity is to be expected between biological sequences from the same species. To focus on ReQuant’s imputation ability we filtered for calls made at sites for which no overlapping 9-mer was trained in the not-imputed model (Fig. 4C and D). ReQuant imputation achieves a marked improvement from 0.85 to 0.97 agreement with Bonito calls at an LLR cutoff of 2, and a sizable increase of calls retained, in k-mer contexts not trained on, from <1% to ∼35%. ReQuant thus enabled reliable modification calling in R10 data for a large proportion of sites that would be discarded by f5c due to a lack of training data.

## Discussion

Directly calling modifications in native DNA is one of the greatest advantages of Nanopore sequencing. However, the translation between raw current values and a DNA sequence with noncanonical bases is hampered by the difficulties of obtaining training data. With the currently existing tools, raw current values for the full repertoire of k-mers must be generated for every modification of interest, and for each version of the kits and pores involved. Synthesizing oligonucleotides covering all permutations is prohibitively expensive, while enzymes are not available for all modification and context combinations. Additionally, enzymatic conversion is not 100% efficient, leading to off-target modifications as well as unmodified targets. This hampers model training by inserting unmodified bases labelled as modified and vice versa in the training set, which negatively influences predictive accuracy.

With the fast development of Nanopore sequencing comes new versions of pores and protocols. At the time of writing, R9.4.1 was still in use, but has now been fully replaced by R10.4.1. The R10 pores’ increased barrel length increases the k-mer size which modifications effect, exponentially increasing the number of context examples required to train and further necessitating ways to train models with incomplete data.

Here we present ReQuant, an algorithm for generalizing the current differences caused by a modification. We show that this generalization is straightforward, summarizing the information of 1280 modified 6-mers in up to 85 “ΔC rules” (Supplementary Fig. S2). This indicates that the effect of a modification is not random in different k-mers but is predictable by its context. ReQuant leverages these consistencies by attempting to extract the ΔC rules from only a fraction of the possible k-mers. We show that this can produce effective methylation models using only 25% of the modified 6-mers for training, and effective CpG glucosylation models using as little as 10% of the modified 6-mers.

For R10 data, we demonstrate that it is possible to train a partial model using the output from Bonito and impute the missing part of the model well enough to accurately call modifications in contexts that were absent from the training data. This means that having a working (DL) model for a limited set of contexts may be enough to create a reliable full model for table-based analyses, extending modification calling capabilities to contexts that would be left untestable otherwise.

Taken together, we show that ReQuant is able to generalize models across various motifs (CpG and GpC), types of modifications (methylation and glucosylation), flow-cell versions (R9 and R10), software packages (Nanopolish and f5c), training data approaches (fully enzymatically modified samples and pre-called partially modified CliveOME samples), and reference genomes (Lambda and human). ReQuant could thus be used to expand the repertoire of accurately detectable DNA modifications. This strategy can be valuable for the detection of DNA lesions, where rare events lead to a noncanonical base, or for experimental setups where noncanonical bases are incorporated under specific conditions.

The computationally ultra-lightweight approach (ReQuant takes < 5 s of wall-clock time for R9 models) saves time and funds otherwise spent on setup, data generation, and training of extensive DL models. Unexpectedly, even when a fully trained model can be created, the approach employed here is able to improve existing models by “denoising” through imputation of the k-mers used for training. This provides a way to improve (table-based) base modification calling with little effort.

One current limitation of our work is the dependence on existing tools. At present, Nanopolish only supports a limited number of motifs and modifications and is limited to R9 flow-cells. While f5c aims to add R10 support, their implementation relies on models that were trained by Nanopolish, thus f5c does not support more motifs. A tool that implements the same algorithms employed by Nanopolish and f5c with more generic support for various motifs would be optimal. Additionally, combinations of modifications are not considered in this work. Theoretically, one may combine the effects of multiple modifications on a k-mer through similar imputations, but reliable train and test data are lacking for now.

ReQuant may prove especially useful for detection of RNA modifications. RNA modifications are common and varied. Thus, models need to be able to cope with multiple different modifications within the same molecule, exponentially increasing the training data requirements. ReQuant might stem this tide of exponential increase in required data.

Aside from providing a tool to impute missing k-mers for improved modification calling, we also sought to improve our understanding of Nanopore data. When comparing modified k-mers with their canonical counterparts, the differences are not random but consistent and even predictable. The current standard approach for the base/modification calling challenge is to use DL-based models that are highly performant but uninterpretable. As a result, it is hard to gauge whether a performance issue is caused by the model, the training setup, experimental issues, or due to a weak effect of modifications on measured current values. For ReQuant, table-based modification calling is an already-proven method (i.e. Nanopolish and f5c) and scatter plots such as presented here allow users to compare the ΔC values and pinpoint the causes of poor performance.

With ReQuant, we show that no black box is needed. Instead, simple, interpretable rules to correct for modifications’ effects on current values are highly performant in all tests we conducted and generalize from just a fraction of the data. This gives ReQuant a clear advantage over current DL approaches. The mechanistic understanding it furnishes allows for further affordable, targeted development of calling uncommon and unnatural modifications from which we can learn and which we can build upon for greater scientific understanding [27]. ReQuant’s sample size efficiency and proven performance make it a valuable tool in any modification calling project.

## Supplementary Material

gkaf323_Supplemental_Files

## Data Availability

The data produced for this study have been deposited in the European Nucleotide Archive (ENA) at EMBL-EBI under accession number PRJEB77524 (https://www.ebi.ac.uk/ena/browser/view/PRJEB77524). The R10 “CliveOME” dataset used in this article was obtained from https://labs.epi2me.io/cliveome_5mc_cfdna_celldna/. Source code for ReQuant and all scripts used to produce figures is available at https://github.com/UMCUGenetics/requant/ and https://doi.org/10.5281/zenodo.15174230.
