# Supplementary material for: ReQuant: improved base modification calling by k-mer value imputation
Source: Nucleic Acids Res. 2025 May 10;53(9):gkaf323. doi: 10.1093/nar/gkaf323 (PMC12065109; doi:10.1093/nar/gkaf323)
Supplement: gkaf323_Supplemental_Files [file gkaf323_supplemental_files.zip › ReQuant_Supplement.pdf]

ReQuant: Improved base modification calling by k-mer value imputation
Supplementary data

Authors:

Roy Straver[1,2], Carlo Vermeulen[1,2], Joe R. Verity-Legg[2,3], Marc Pagès-Gallego[1,2], Dieter G.G. Stoker[1,2], Alexander van Oudenaarden[2,3] and Jeroen de Ridder[1,2]

Affiliations:

[1]Center for Molecular Medicine, University Medical Center Utrecht, 3584 CX Utrecht, The Netherlands.
[2]Oncode Institute, 3521 AL Utrecht, The Netherlands
[3]Hubrecht Institute-KNAW (Royal Netherlands Academy of Arts and Sciences), 3584 CT Utrecht, The Netherlands.

Modification calling

For modification calling the raw currents of each read are first basecalled to canonical base sequences and mapped to a reference genome. Using a Dynamic Programming approach such as Dynamic Time Warping, the raw current signal is aligned to the reference sequence. Examples of raw currents aligned to a reference base sequence are shown in Fig. 1A. Using this raw-to-base alignment the raw current data around the base motif is extracted and fed to the modification callers. Modification callers then classify the measured currents as more likely to be caused by a canonical (unmodified) or modified motif.

Remora/Megalodon

DL models are ostensibly well suited for the task of modification calling: linking modification status to a raw current signal is a highly complex function that DL models excel at learning. However, the best self-trainable methylation calling toolset at the time of writing, Remora/Megalodon, does not generalize well to unseen sequence contexts. To demonstrate this, we trained Remora R9 models with various percentages of missing 6-mer examples. To create this incomplete training data, we divided the Lambda reference genome into smaller parts (sections), each containing roughly a target percentage of modified 6-mers. For each section, we trained a Remora model with the read parts covering CpG contexts in that section. We then tested each model on read parts covering CpG contexts in the rest of the Lambda genome, outside of the training section. The Remora trained models were used to call modifications with Megalodon. As shown in Fig. 1B, Remora's accuracy drops tremendously and continuously during serial removal of percentages of 6-mers from the training set, while this has little effect on the frequency of calls discarded due to low call-certainty values (Supplementary Fig. S20).

Table-based methods

Nanopolish, Tombo, and similar approaches assume six bases in the pore at a time measurably influence the current for Nanopore's R9 flow-cells. Any two 6-mers, measured sequentially in 1 base-pair step sliding windows, overlap by five bases. Thus, each base that transits the pore is measured six times in six different 6-mers. Table-based modification callers use a table to describe the raw currents they expect for each canonical 6-mer. This table is extended to include a modified base ("M") as a fifth possible "base" and the model is trained to determine the expected currents for all possible 6-mers with the modification. For modification calling, the measured current sequence of each 6-mer containing a potentially modified motif is compared to the expected raw values of both its canonical and modified alternatives (Fig. 2A). Using an HMM or statistical methods, a call is made using a Log Likelihood Ratio (LLR) between the two options. In contrast to DL, the 6-mer tables as employed by Nanopolish and Tombo models are human-interpretable. However, these methods have a severe limitation: if a modified 6-mer was not in the training data, it cannot be called reliably as its value is simply unknown.

Bare minimum training set

We showed that ReQuant performs well when having seen only 25% of randomly selected k-mers. We hypothesised that a minimal k-mer set from which ReQuant can reliably impute exists, most likely one where every possible base at every position in the k-mer is covered equally. To test this, we selected context pairs from the lambda genome with a single target motif, where no base-motif combination in the k-mers overlapped, taking into account both forward and reverse complementary context versions. Every pair of contexts selected thus adds an example for each possible base at each position for every motif location in the k-mer. As we train both forward and reverse stranded sequence contexts of a marked location, we effectively add two contexts per marked site, and we should be able to cover all base-motif combinations by marking just two sites. Multiple such pairs are combined to create various levels of coverage for each base in the k-mers, but pairs are only selected if their k-mers are not already covered by a previous context pair, ensuring each minimal context set covers the most k-mer variation possible. In this regime, improvements in call rates stabilise around a coverage of 8 to 10 per base, equalling 160 to 200 k-mers, or about 12.5 to 16% of all modified k-mers covered (Supp. Figure S9a). The LLR based discarded call rates for the canonical dataset shown in Supp. Figure S9b suggest a similar stabilisation. A coverage of 10 per base is obtained from 20 sites where both forward and reverse stranded reads are used for training, totalling 40 context sequences to train from. The results at 16 coverage (~25% of modified k-mers) resemble the results previously obtained for *T*=25% k-mers by random picking, suggesting this optimised context selection does not significantly improve over total random context selection. Hence, at *T*=25% most bases are likely covered well enough, assuming no significant lack of a particular base at any position in the k-mer.

Nanopolish edits

For GpC contexts we also changed the alphabet in the output model file to match:

```
#alphabet gpc
```

When attempting to train a GpC context model this provided an error:

```
nanopolish: src/common/nanopolish_alphabet.h:145: virtual std::string Alphabet::reverse_complement(const string&) const: Assertion `str[i] != 'M'' failed.
```

Which was resolved by commenting out the offending assertion, which appears to be a sanity check that fails as the GM context may now have a single M at the other end of a k-mer compared to MG motifs.

Supplementary figures

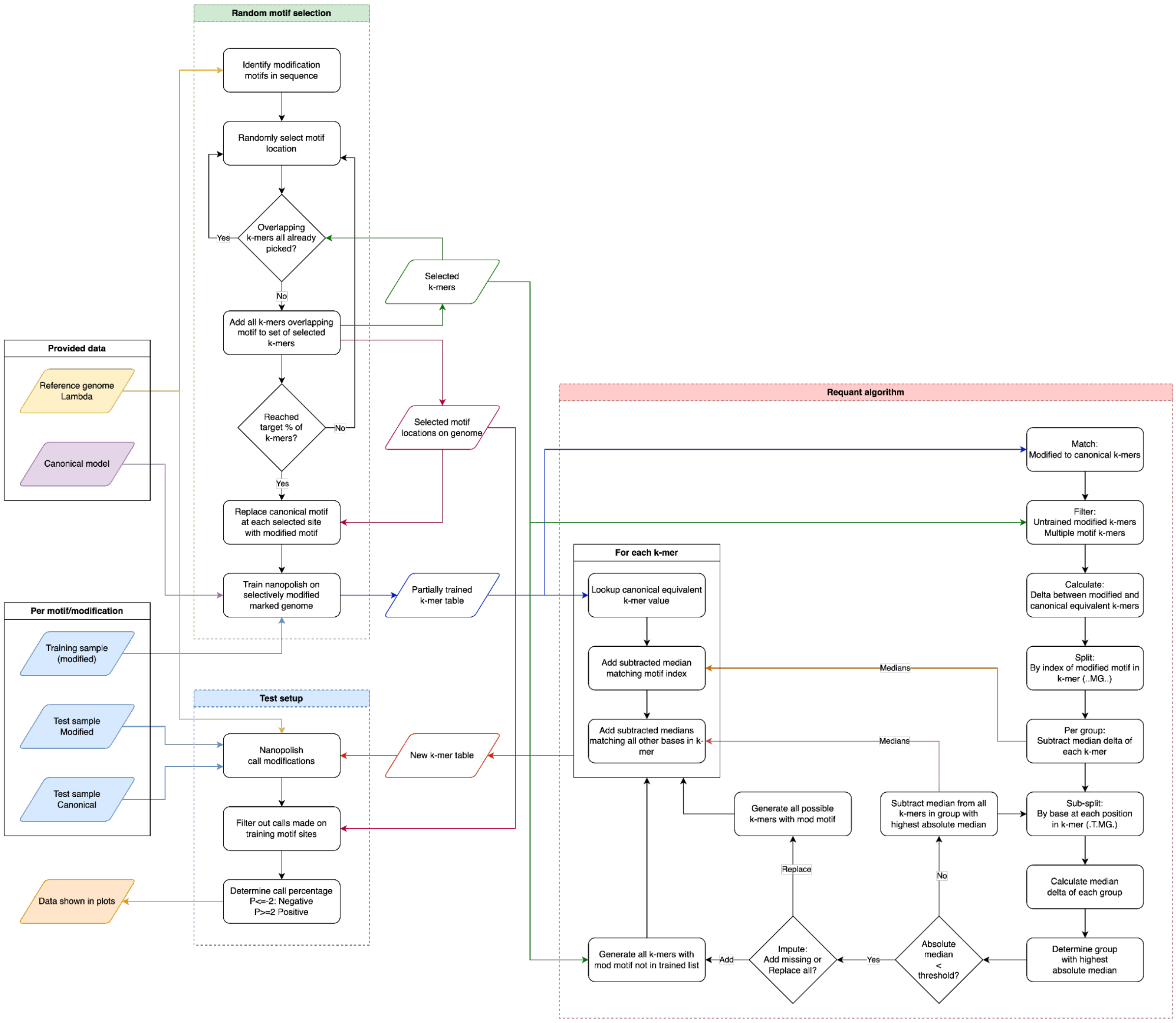

Figure S1: ReQuant's algorithm presented in a diagram. Any colours are only to enhance visualisation of where data is used while the logical order of steps is shown in black. Data required is shown on the left, with reference genome and a canonical model as assumed obtainable without further work. Underneath are the different samples used for training and testing per motif and modification. Training logic is shown in the top left green section, taking a reference genome and canonical model plus an example sample with the target modification. Our algorithm is shown in the red section on the right, taking the partially trained k-mer table and information on what k-mers were trained to produce a new k-mer table. The test setup, outlined in blue in the bottom left, shows the modification calling and output filtering for each type of sample.

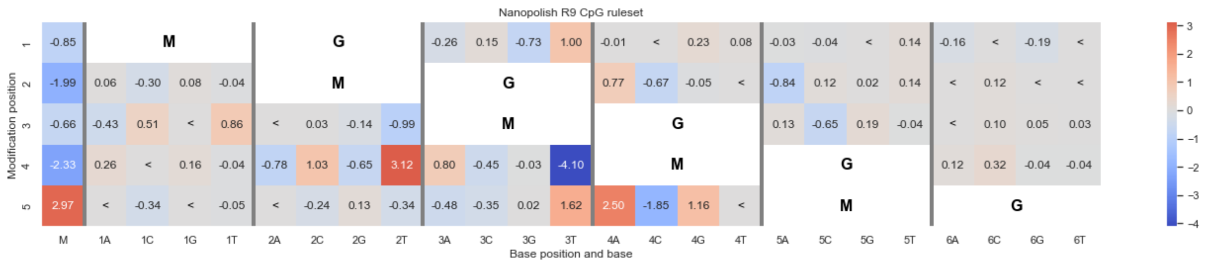

Figure S2: All *Δ*C rules CpG model as determined by ReQuant shown in a heatmap. Colours indicate the value, which is also annotated in each square. Rows indicate the position of the MG motif in the 6-mer (e.g. 1 means MGNNNN, 2 means NMGNNN, etc.). The first column shows the rule for having a MG motif in the position indicated by the rows, while all other columns indicate rules specific for having a base (ACGT) at any other position in the 6-mer. Vertical grey lines were added to group the bases at each position in the 6-mers. Light grey areas with a < indicate no rule was determined for a combination. While example 6-mers existed, the delta they incurred was below the threshold set for ReQuant to stop. The MG motif position follows a diagonal from top left to bottom right, their corresponding values are already determined by the motif position specific rules in the first column, hence are left empty. Note that for each position of MG there are 4 possible bases, + the MG motif itself = (1+4*4) * 5 = 85 possible *Δ*C rules.

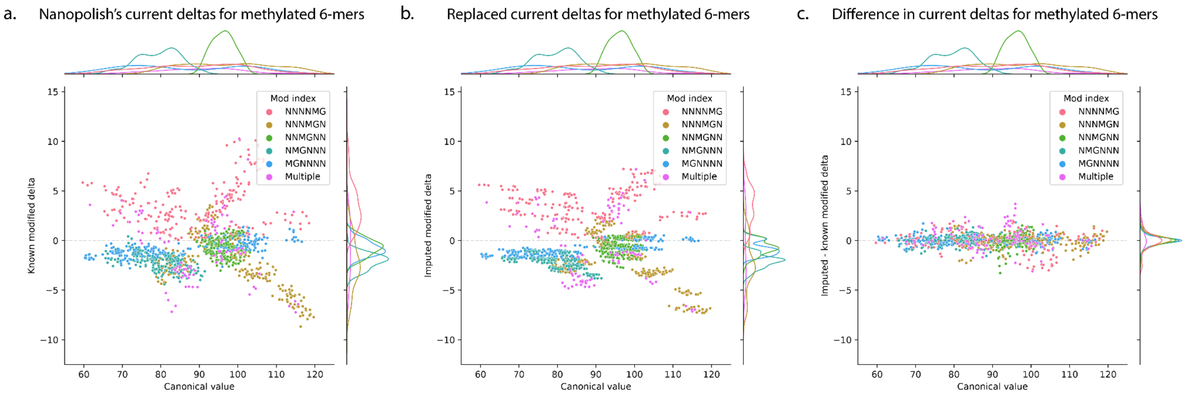

Figure S3: a. Plot showing the values and distributions of the canonical equivalent of a modified 6-mer (x-axis) and the delta between this canonical value and the modified 6-mers value (y-axis), using the built-in CpG methylation model for Nanopolish, split and coloured by location of the modified base in the 6-mers. b. Similar to a, but showing the model after replacing values of all 6-mers with a modified base with imputed values. The 6-mer grouping behaviour in the output model has a similar distribution to the original (a). c. Similar to a,b but the y-axis now shows the difference between the trained and the imputed value for each modified k-mer, this is the difference between plots a and b. The narrow band on the y-axis with all k-mers suggests the differences between the original and the imputed model are small.

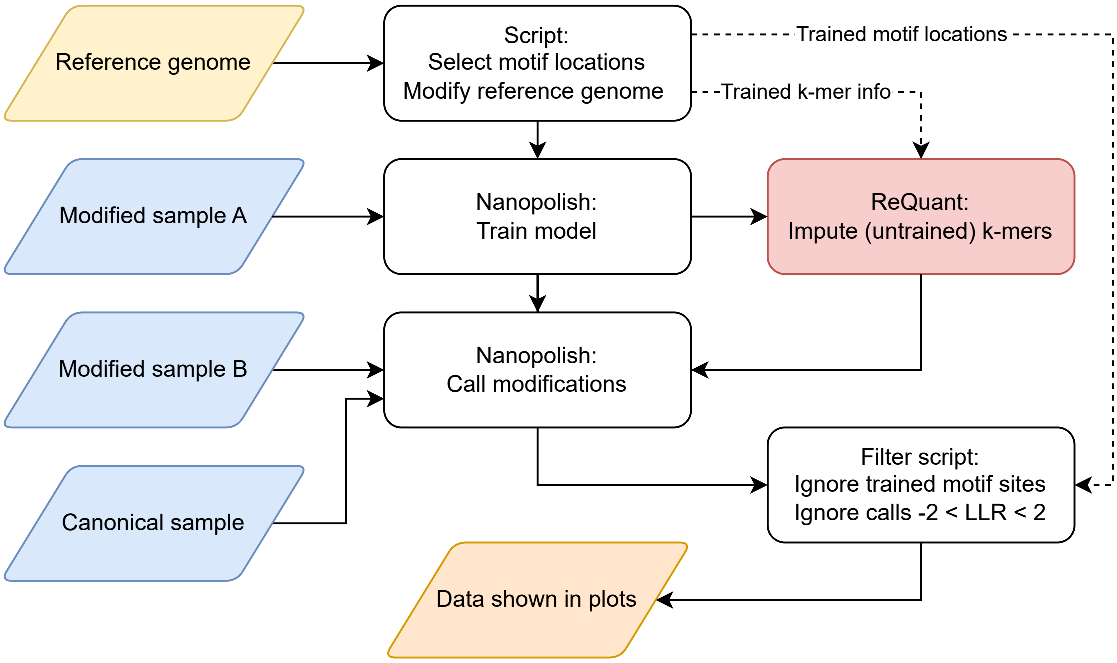

Figure S4: Overview diagram of our training setup with ReQuant marked in red. A new modification/motif combination requires modified samples A and B, while the canonical sample can be reused for testing each modification. A canonical model is included in Nanopolish hence no canonical training data is required.

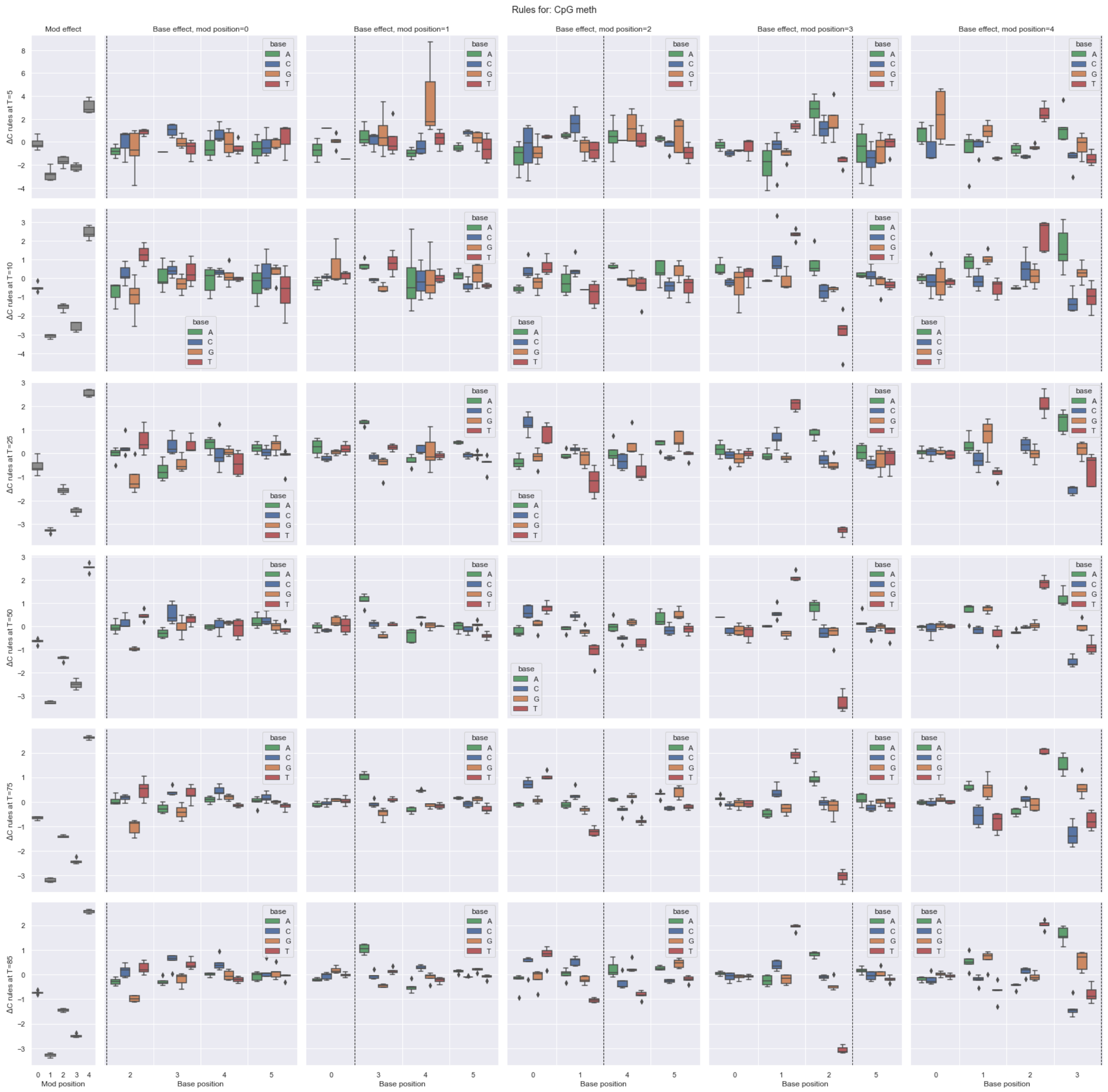

Figure S5: Boxplots showing the *Δ*C rule distributions over various models. Every row shows the rules obtained over various models at a percentage (*T*) of 6-mers used for training. The first column shows the rules for having a methylated CpG motif at each position in the 6-mer (MGNNNN, NMGNNN, etc.). The other five columns show the rules specific for having a methylated CpG at a position combined with a particular base in another position. Four groups indicate the location of the base (separated by white vertical lines) and the base itself (coloured boxes, ordered ACGT within the groups). The methylated CpG position is indicated by the black dashed vertical line. For example, at *T*=85% (bottom row), mod position=3 (second column from the right), at base position=2 (third group of 4), T (red, the fourth box in the group), indicates the effect of a methylated 6-mer that matches NNTMGN is usually about -3. The vertical spread of values in each plot decreases when the training set (*T*) increases, indicating the *Δ*C rules approach a specific, stable, set of values.

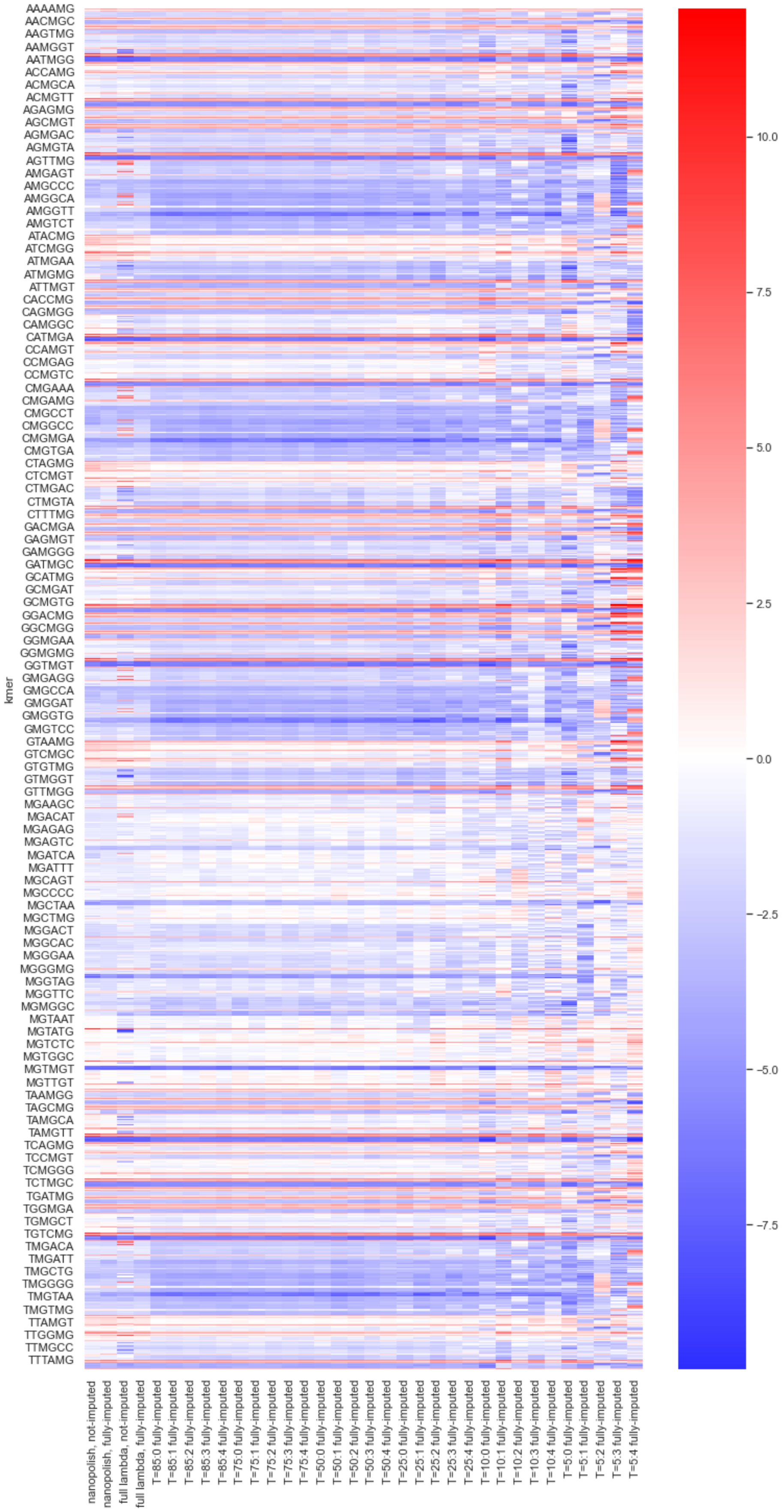
kmer
AAAAMG
AACMGC
AAGTMG
AAMGGT
AATMGG
ACCAMG
ACMGCA
ACMGTT
AGAGMG
AGCMGT
AGMGAC
AGMGTA
AGTTMG
AMGAGT
AMGCCC
AMGGCA
AMGGTT
AMGTCT
ATACMG
ATCMGG
ATMGAA
ATMGMG
ATTMGT
CACCMG
CAGMGG
CAMGGC
CATMGA
CCAMGT
CCMGAG
CCMGTC
CMGAAA
CMGAMG
CMGCCT
CMGGCC
CMGMGA
CMGTGA
CTAGMG
CTCMGT
CTMGAC
CTMGTA
CTTTMG
GACMGA
GAGMGT
GAMGGG
GATMGC
GCATMG
GCMGAT
GCMGTG
GGACMG
GGCMGG
GGMGAA
GGMGMG
GGTMGT
GMGAGG
GMGCCA
GMGGAT
GMGGTG
GMGTCC
GTAAMG
GTCMGC
GTGTMG
GTMGGT
GTTMGG
MGAAGC
MGACAT
MGAGAG
MGAGTC
MGATCA
MGATTT
MGCAGT
MGCCCC
MGCTAA
MGCTMG
MGGACT
MGGCAC
MGGGAA
MGGGMG
MGGTAG
MGGTTC
MGMGGC
MGTAAT
MGTATG
MGTCTC
MGTGGC
MGTMGT
MGTTGT
TAAMGG
TAGCMG
TAMGCA
TAMGTT
TCAGMG
TCCMGT
TCMGGG
TCTMGC
TGATMG
TGGMGA
TGMGCT
TGTCMG
TMGACA
TMGATT
TMGCTG
TMGGGG
TMGTAA
TMGTMG
TTAMGT
TTGGMG
TTMGCC
TTTAMG
nanopolish, not-imputed
nanopolish, fully-imputed
full lambda, not-imputed
full lambda, fully-imputed
T=85:0 fully-imputed
T=85:1 fully-imputed
T=85:2 fully-imputed
T=85:3 fully-imputed
T=85:4 fully-imputed
T=75:0 fully-imputed
T=75:1 fully-imputed
T=75:2 fully-imputed
T=75:3 fully-imputed
T=75:4 fully-imputed
T=50:0 fully-imputed
T=50:1 fully-imputed
T=50:2 fully-imputed
T=50:3 fully-imputed
T=50:4 fully-imputed
T=25:0 fully-imputed
T=25:1 fully-imputed
T=25:2 fully-imputed
T=25:3 fully-imputed
T=25:4 fully-imputed
T=10:0 fully-imputed
T=10:1 fully-imputed
T=10:2 fully-imputed
T=10:3 fully-imputed
T=10:4 fully-imputed
T=5:0 fully-imputed
T=5:1 fully-imputed
T=5:2 fully-imputed
T=5:3 fully-imputed
T=5:4 fully-imputed
10.0
7.5
5.0
2.5
0.0
−2.5
−5.0
−7.5

Figure S6: Heatmap showing the differences between the current values for 6-mers with a methylated CpG motif and their equivalent canonical 6-mers. The modified 6-mers are shown along the y-axis, their annotations are subsampled due to limited vertical space. Along the x-axis are various models, sorted by their training 6-mer set size. The first models on the left are the CpG model included with Nanopolish, the same Nanopolish model after ReQuant imputed all values, a model trained on all CpG sites in our Lambda training data, and a ReQuant imputed version of that fully trained model. While the not-imputed full-lambda model (column 3) looks quite different from the other models, after imputation (column 4) it is nearly identical to the full-imputed version of Nanopolish' included CpG model (column 2). All sub-set trained models (columns 5 and beyond) appear more coloured, suggesting these models all show a larger difference between the modified and their equivalent canonical 6-mers. This is not a difference introduced by ReQuant, as the full lambda fully-imputed model appears at similar intensity as Nanopolish' included models. This difference is likely introduced by the sub-sampling method employed, where all CpG sites are methylated but only a subset of sites is marked as such, causing Nanopolish' internal normalisation and matching of raw currents to the expected values to make consistent, slight, errors.

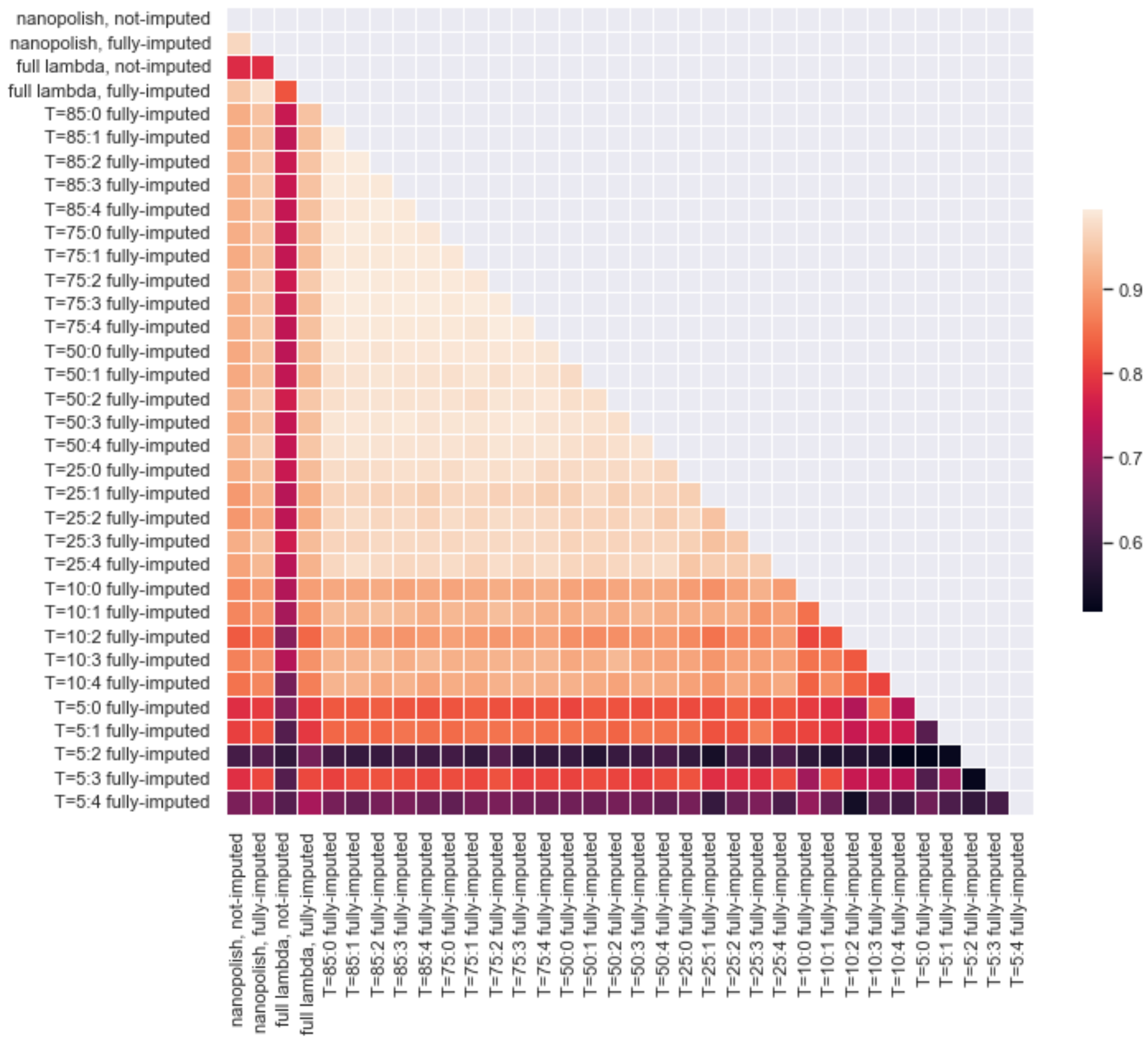

Figure S7: A heatmap showing correlation (colour) among all models shown in Supplementary Fig. S6, based on the 6-mer values shown in Supplementary Fig. S6. All models from *T*=25 up to and including *T*=85 show very strong correlations, suggesting that indeed the models produced at *T*=25 would perform similar to models based on more training 6-mers. Models produced from training sets at *T*=5 vary quite a bit, as indicated by the lower correlation scores among each other and to models with more 6-mers included for training. As in Supplementary Fig. S6, the full lambda not-imputed model also stands out as not correlating very well with anything, but the full lambda fully-imputed model created from it does correlate really well (r=0.98) with the Nanopolish fully-imputed model.

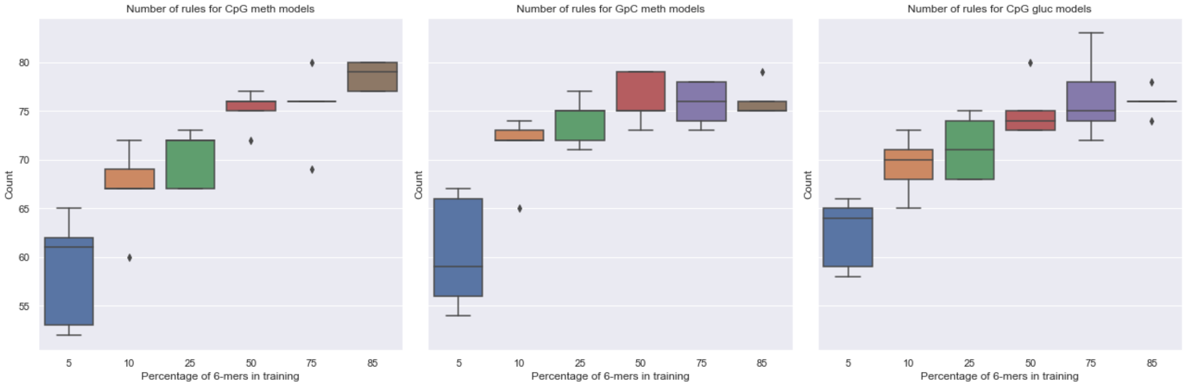

Figure S8: Boxplots showing the number of rules (y-axis) obtained for various models at different settings of *T* (x-axis), for each dataset (CpG methylated, GpC methylated, GpC glucosylated). The number of rules appears largely dictated by the size of the 6-mer training set, as ReQuant does not determine rules for modification-base combinations that do not occur in the training data.

a.

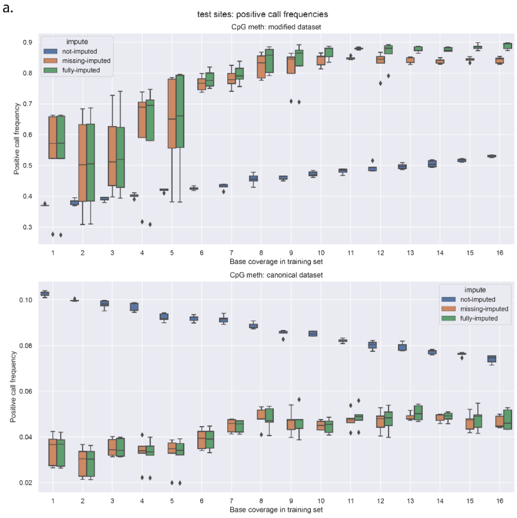

cover	kmers	%
1	20	1.56
2	40	3.13
3	60	4.69
4	80	6.25
5	100	7.81
6	120	9.38
7	140	10.94
8	160	12.50
9	180	14.06
10	200	15.63
11	220	17.19
12	240	18.75
13	260	20.31
14	280	21.88
15	300	23.44
16	320	25.00

b.

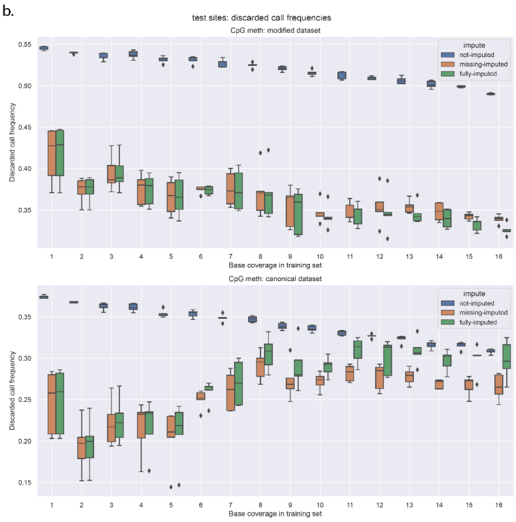

Figure S9: a. Boxplots showing positive call rates analogous to previous boxplots for the minimal training set approach. The x-axis now shows coverage per base per motif position, where the value indicates how many k-mers in the training set covered each base at each position in the k-mer. Each step equals 20 k-mers, or about 1.56% in previous boxplots, as shown in the table. b. Similar to a, but now the data shows the discarded call frequencies when filtering for calls with an absolute LLR of at least 2.

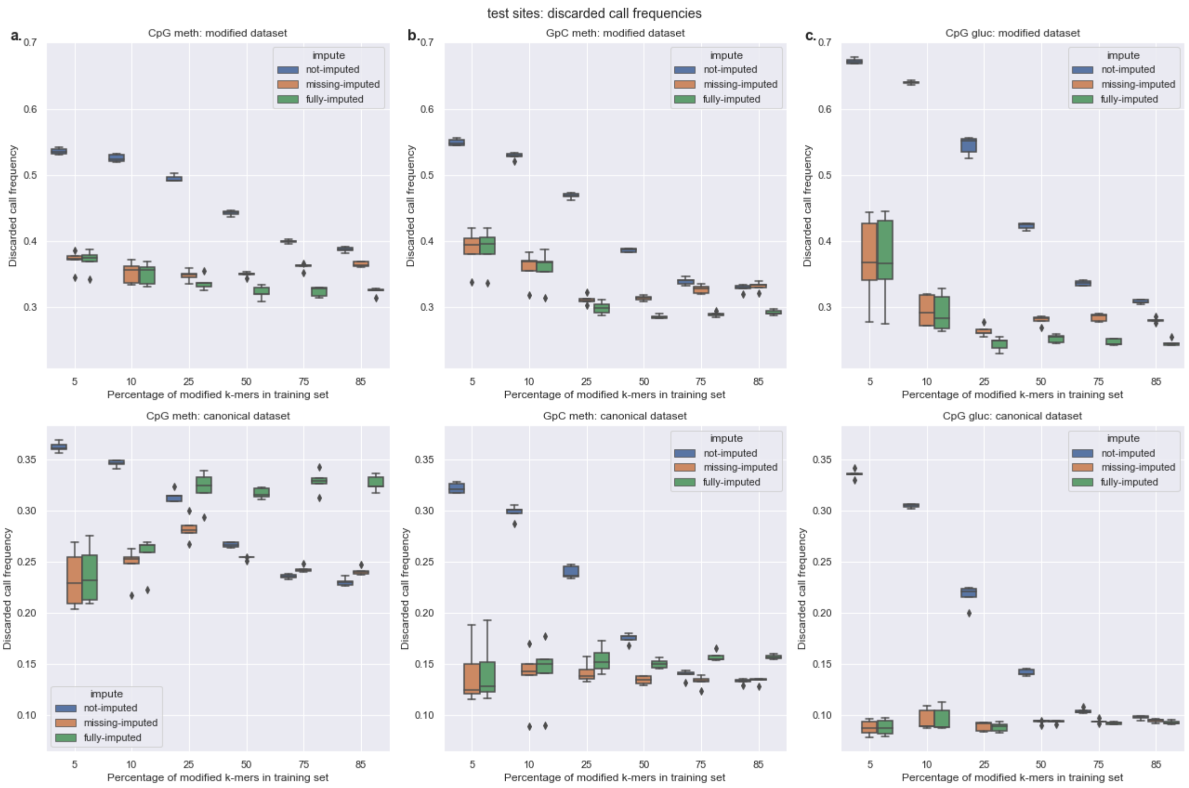

Figure S10: Nanopolish provides a Log-Likelihood Ratio (LLR) per site and suggests using a cutoff to filter out uncertain calls, removing calls with an LLR between -2 and 2. a. Discarded call frequencies for CpG methylation data. The increase in correct call rates shown in Figure 3 is not due to making more uncertain calls, which could unfairly inflate correct call rates. The *not-imputed* method clearly suffers from the lack of trained k-mers. The *fully-imputed* method has more uncertain calls at higher training percentages in the canonical sample. b. Discarded call frequencies for GpC methylation data. c. Discarded call frequencies for CpG glucosyl data.

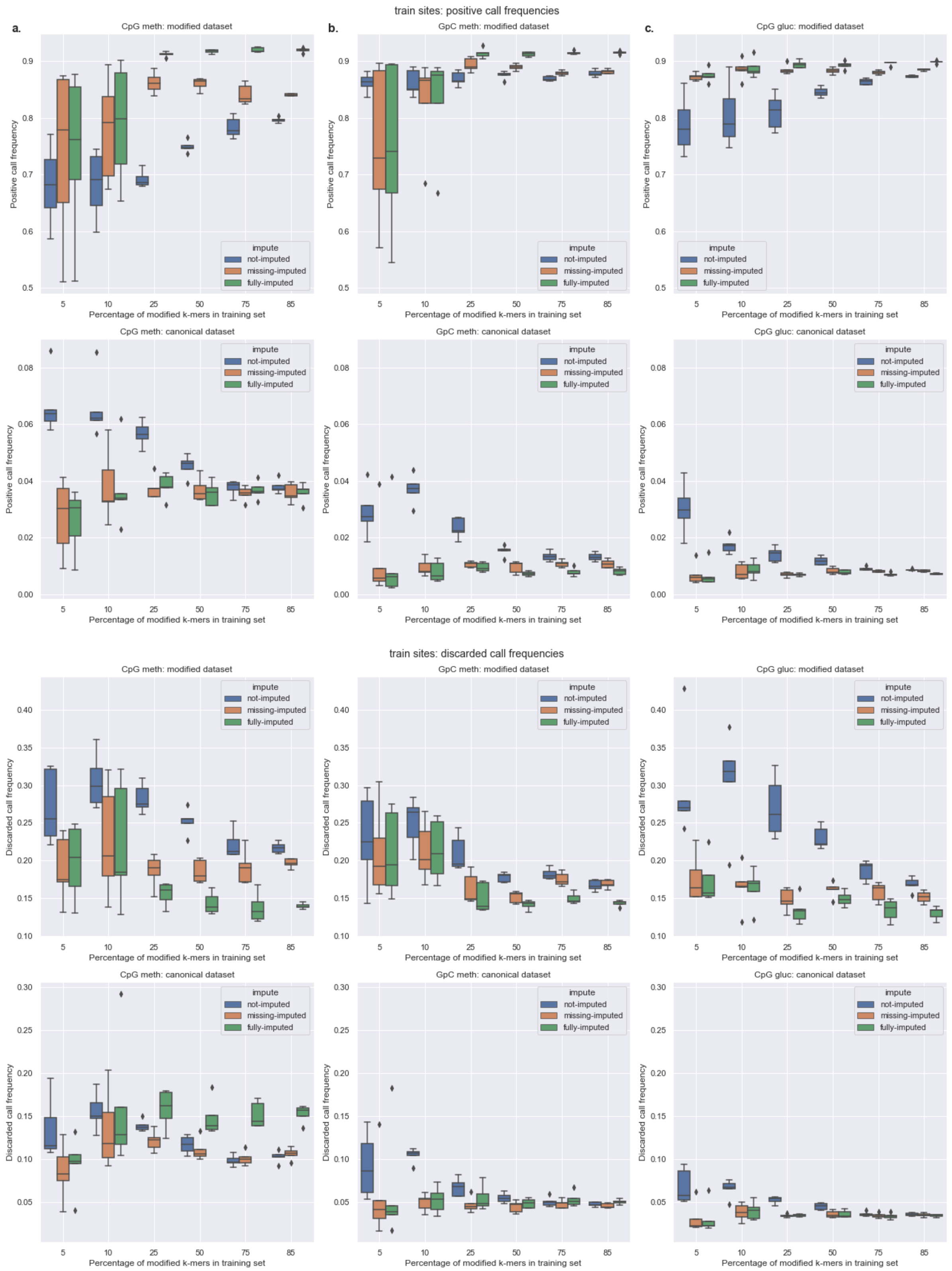

Figure S11: Boxplots showing positive call rates for various positive (top) and the negative (second row) datasets, similar to Figure 3, but only calls made on sites that were included for training the models are now counted. The bottom 2 rows include the frequencies of calls being discarded, similar to Supplementary Fig. S10. Differences in call rates between *not-imputed* and *missing-imputed* can be explained by Nanopolish' behaviour of extending the region around a motif site until no more motif is within a 10 base range before calling. Unobserved sites nearby trained sites thus influence these numbers.

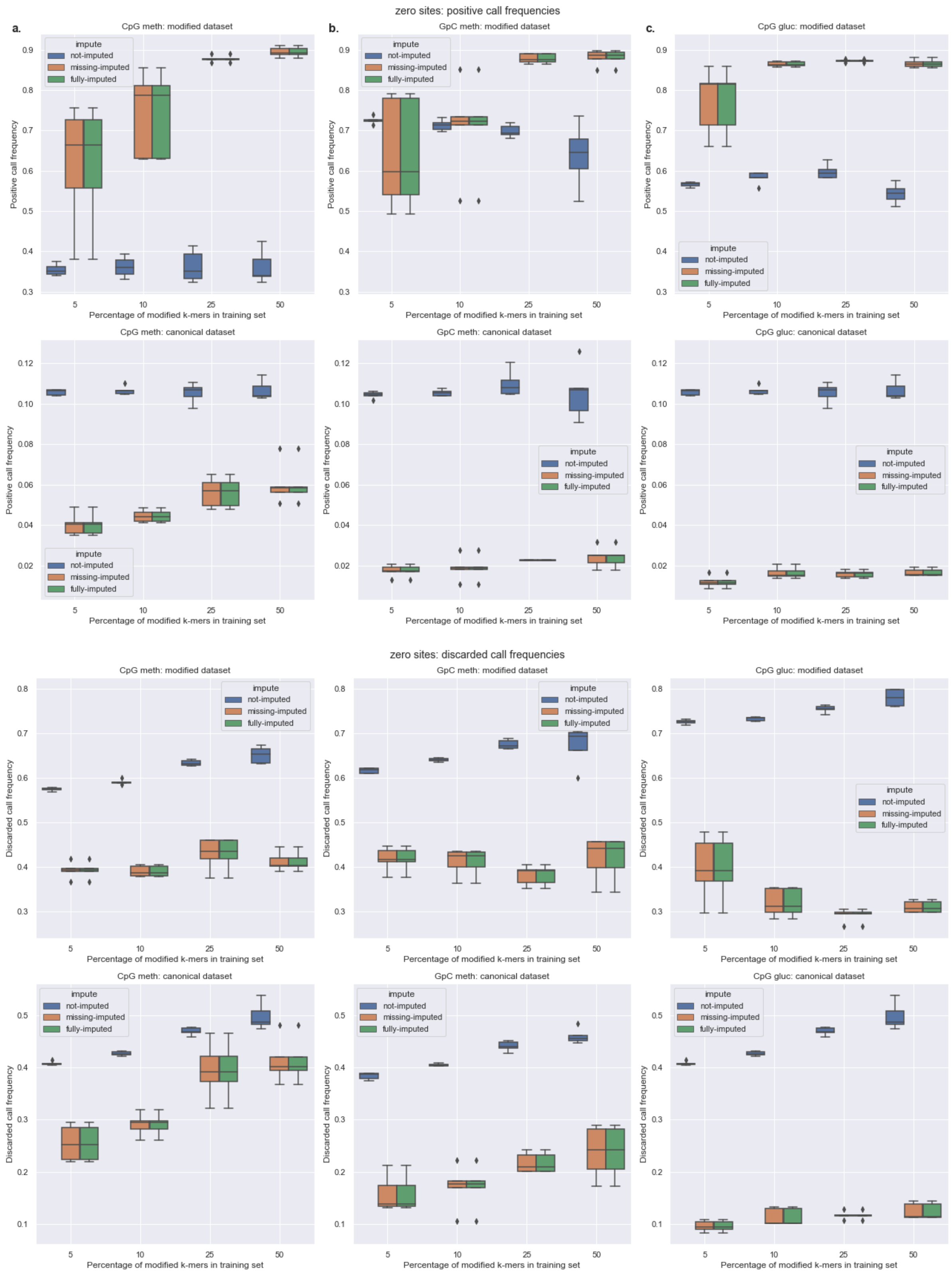

Figure S12: Boxplots showing positive call rates for various positive (top) and the negative (second row) datasets, similar to Figure 3, but only calls made on sites without any 6-mer overlapping the training data are now counted. The bottom 2 rows include the frequencies of calls being discarded, similar to Supplementary Fig. S10.

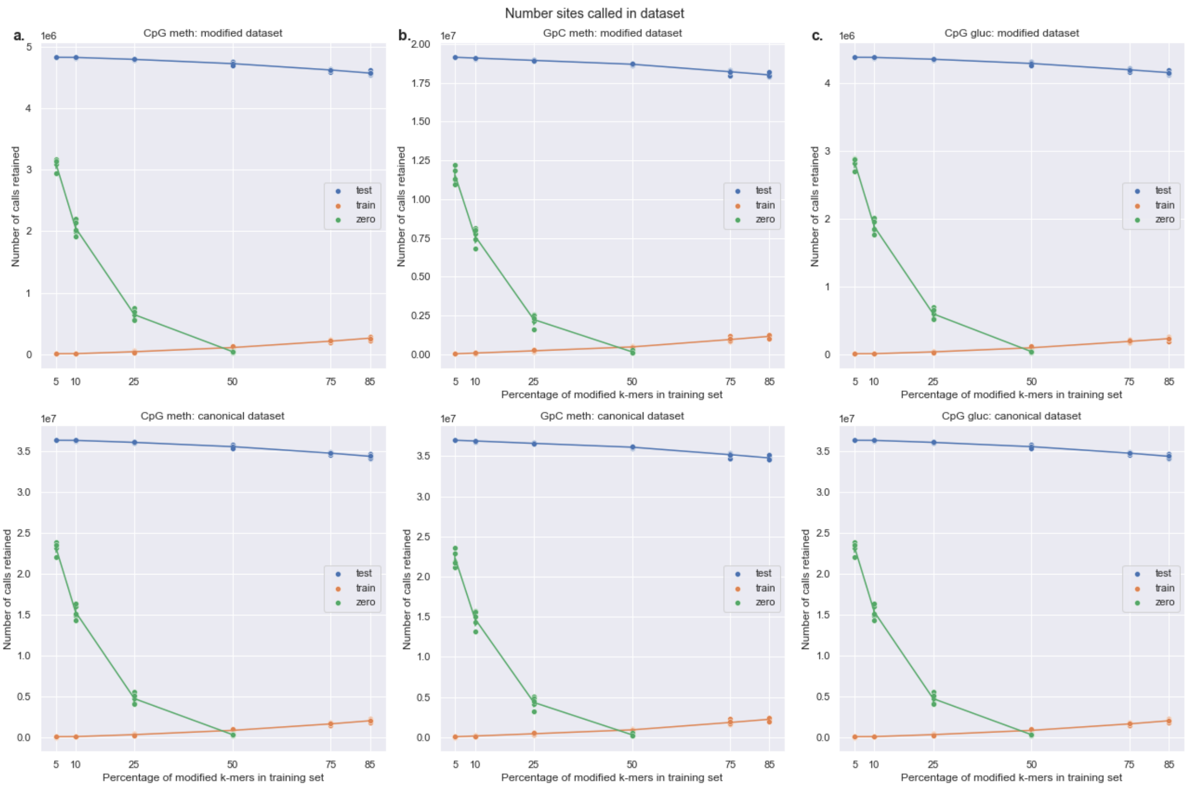

Figure S13: For all models the number of sites included to make Figure 3 (blue), Supplementary Fig. S11 (orange) and Supplementary Fig. S12 (green). Both CpG meth and CpG gluc tests used the same CpG sites for training, hence a and b show the same data points. As each CpG site in the test set is overlapped by five k-mers, the number of sites in the test set that do not overlap with any k-mers in the training set (the zero set) decreases dramatically as more k-mers are included for training and little to no calls were recorded for *T*>50%.

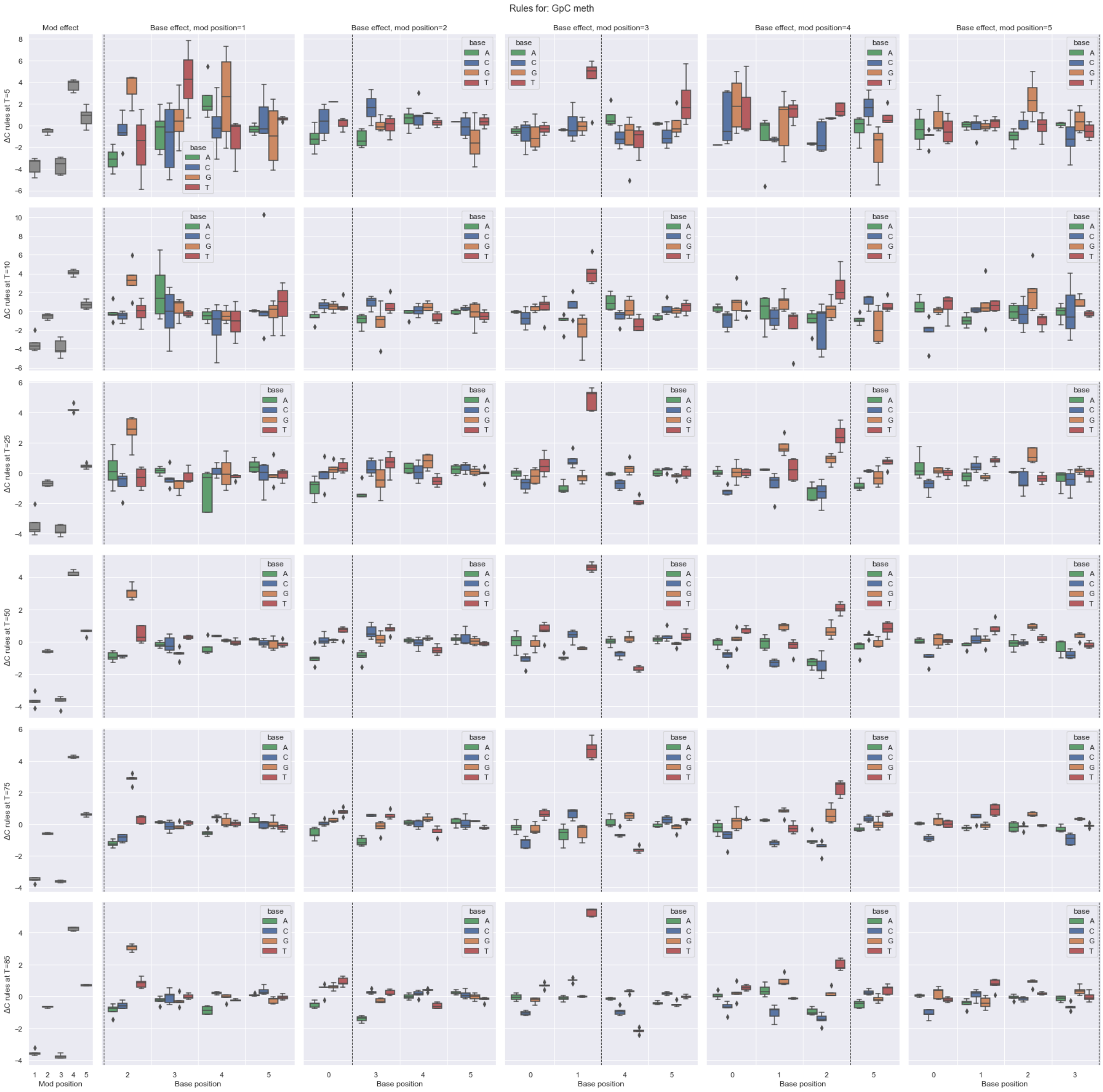

Figure S14: Boxplots analogous to Supplementary Fig. S5, but for GpC methylated data, showing the *Δ*C rules for 6-mers with a methylated GpC motif.

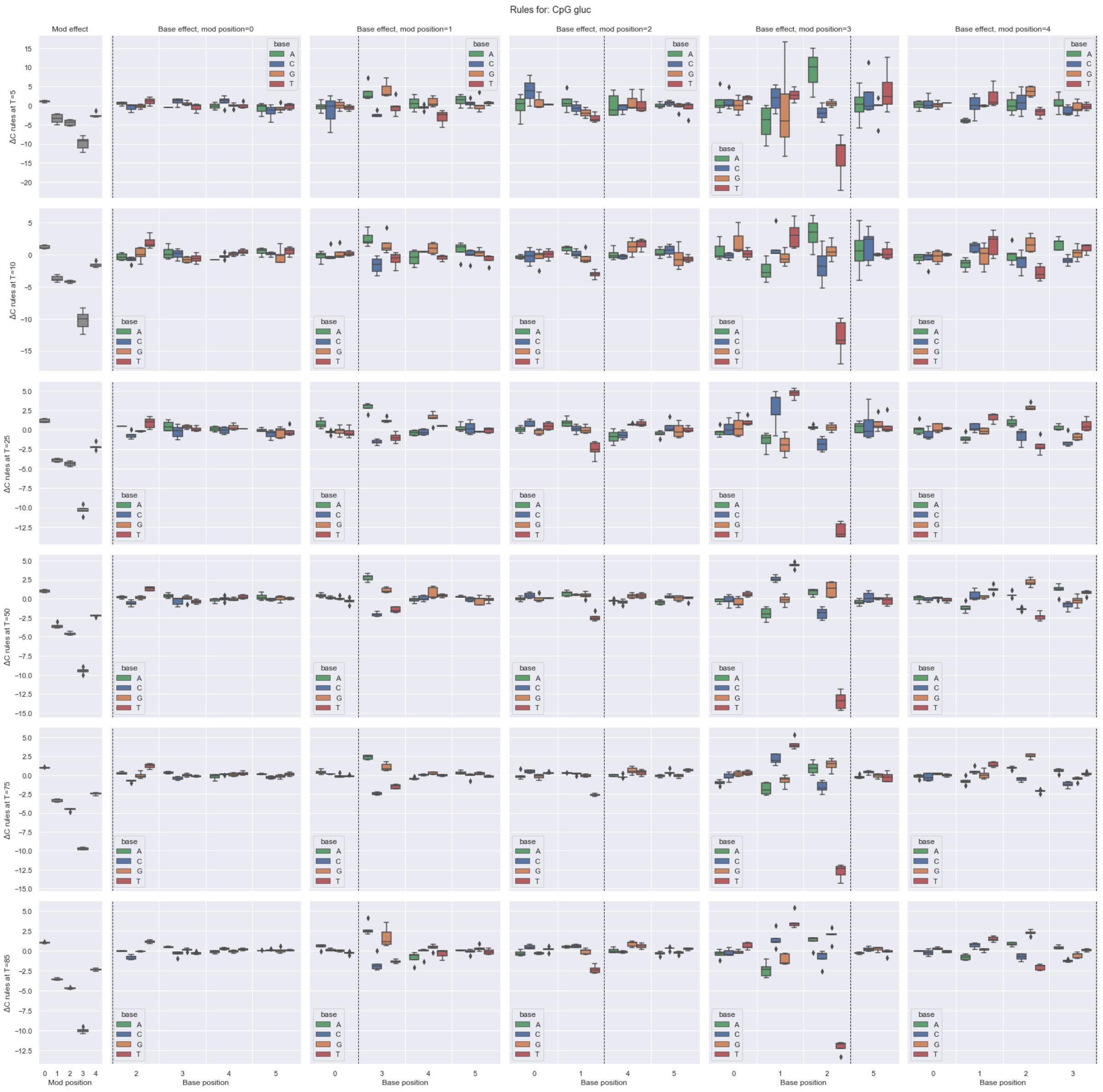

Figure S15: Boxplots analogous to Supplementary Fig. S5 and S14, but for CpG glucosylated data, showing the *Δ*C rules for 6-mers with a glucosylated CpG motif. Note the y-axis scale changed to accommodate for the much larger *Δ*C rule values, showing the much larger effect of glucosyl on the raw Nanopore currents than methylation.

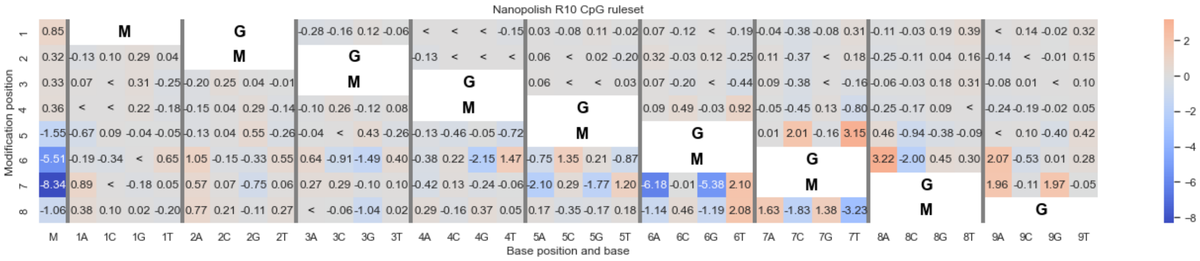

Figure S16: Heatmap of the R10 CpG model, similar to Supplementary Fig. S2.

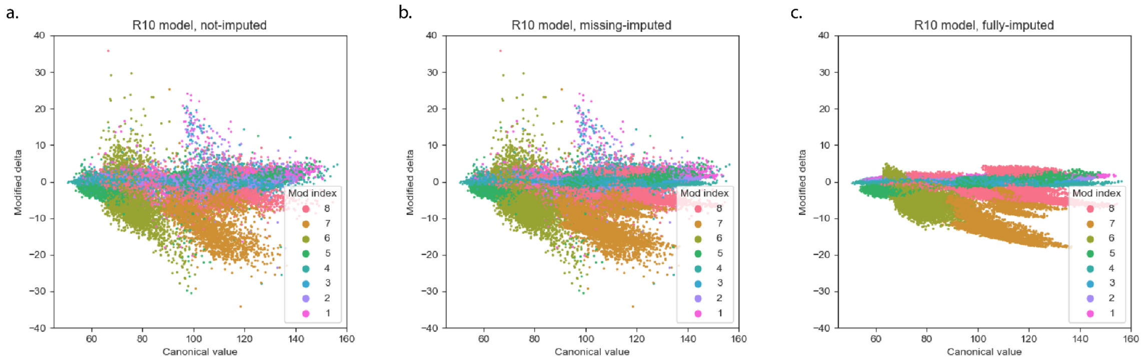

Figure S17: Scatter plots comparing canonical k-mer values (x-axis) to modified k-mer values (y-axis) using 9-mer CpG models for R10. The Mod index in the legends refers to the position of the modification in the 9-mers, e.g. index 3 means NNMGNNNNN. a. Trained on chr22 covering ~36% of all possible 9-mers. b. The same model as a. but unobserved k-mers have been imputed by ReQuant. c. The same model as b. but trained k-mers were also replaced by values imputed by ReQuant. Note the reduced vertical spread of points in c compared to a and b. Considering the limited amount of data used for training the model we assume the spread was originally caused by mistraining 9-mers, making the model look noisy.

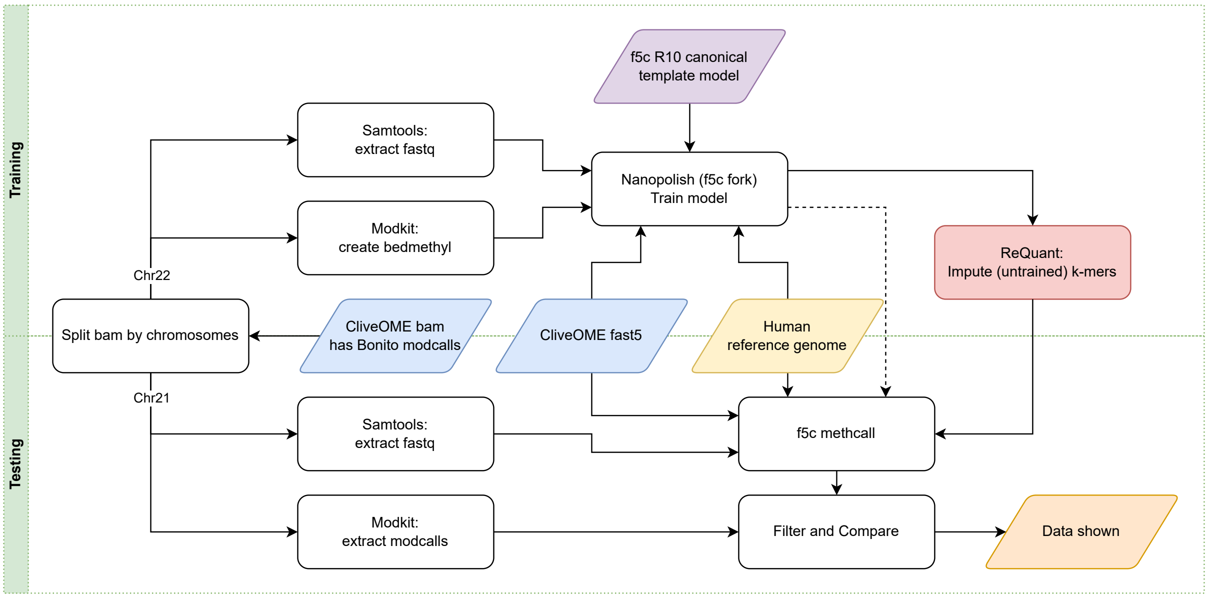

Figure S18: Schematic overview of the R10 train and test setup. Data is split into a train (chromosome 22, upper half) and test (chromosome 21, lower half) set. For both sets, we extracted the included modification calls (made using the Bonito Deep Learning methylation caller) using *modkit* to serve as our ground truth. We used the f5c modification caller (An alternative to Nanopolish that performs raw current value alignment and modification calling; See R10: f5c in Materials and Methods in the main text). We trained a not-imputed model on chromosome 22 (covering ~36% of all possible modified 9-mers). Then, we applied ReQuant to this first k-mer table to get a *missing-imputed* and *fully-imputed* model. We then tested the models trained on chromosome 22 on chromosome 21 (both on all k-mers and only those unseen on chromosome 22 during training) to verify that ReQuant imputation also works for R10 pores.

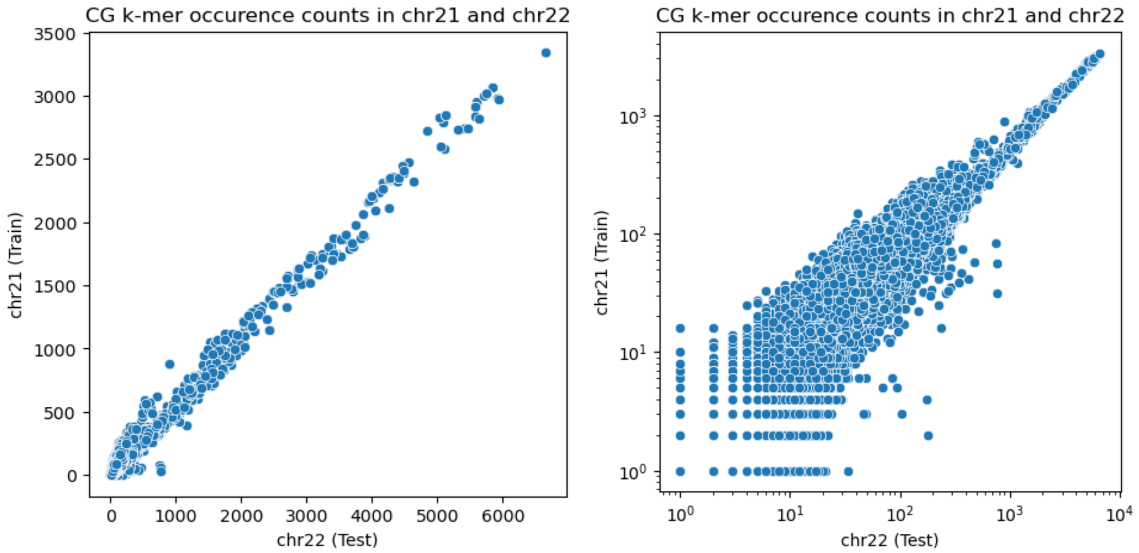

Figure S19: Occurrence rate per 9-mer with CpG motif in chromosome 22 (x-axis) vs in chromosome 21 (y-axis). Plotted linearly (left) and logarithmically (right).

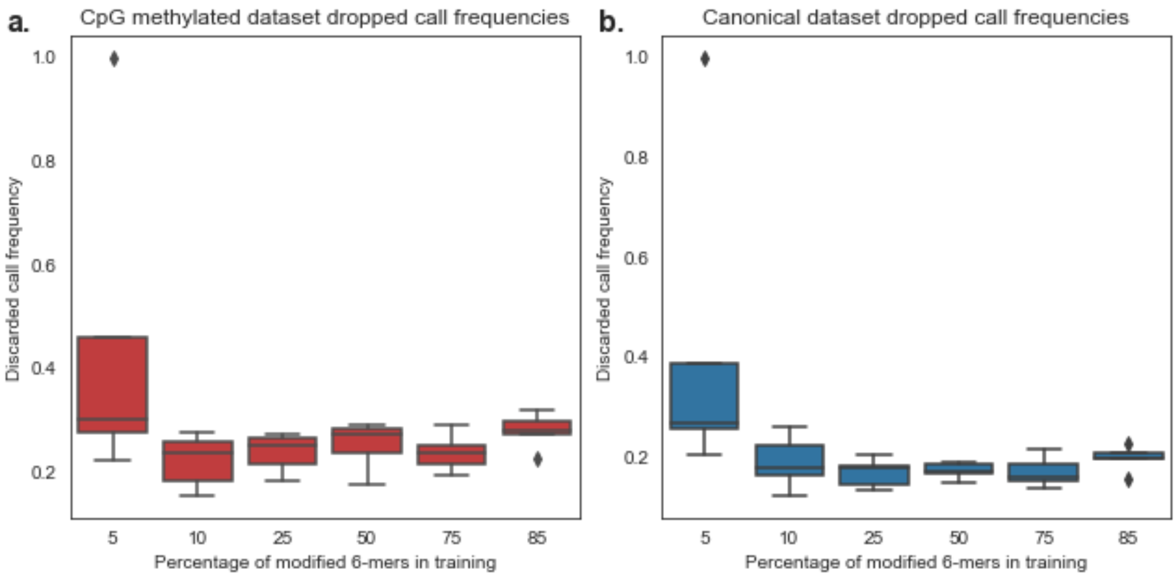

Figure S20: Discarded call frequencies for Remora/Megalodon models trained on sections of the lambda genome, and tested on the rest of the lambda genome. A cutoff was applied for both positive (>=1) and negative (<=-1) calls, meaning any call with an LLR between -1 and 1 would be discarded due to the model being uncertain.
